# Identification and Validation of Tissue-Specific Housekeeping Markers for the Amazon River Prawn *Macrobrachium amazonicum* (Heller, 1862)

**DOI:** 10.3390/genes17010026

**Published:** 2025-12-28

**Authors:** Gabriel Monteiro de Lima, Mônica Andressa Leite Rodrigues, Rômulo Veiga Paixão, Ítalo Lutz, Manoel Alessandro Borges Aviz, Janieli do Socorro Amorim da Luz Sousa, Bruna Ramalho Maciel, Luciano Domingues Queiroz, Carlos Murilo Tenório Maciel, Iracilda Sampaio, Eduardo Sousa Varela, Cristiana Ramalho Maciel

**Affiliations:** 1 Instituto de Estudos Costeiros, Universidade Federal do Pará, Campus Universitário de Bragança, Al. Leandro Ribeiro, s/n, Bragança 68600-000, Pará, Brazil; gabriel.lima@braganca.ufpa.br (G.M.d.L.);; 2 Embrapa Pesca e Aquicultura, Av. NS 10, Cruzamento com a Av. LO 18 Sentido Norte Loteamento-Água Fria, Palmas 77008-900, Tocantins, Brazil; 3 Instituto Federal de Educação, Ciência e Tecnologia do Pará, Campus Cametá, Av. Euclides Figueiredo, s/n, Cametá 68400-000, Pará, Brazil; 4 Instituto Federal de Educação, Ciência e Tecnologia do Pará, Campus Tucuruí, Av. Brasília, s/n, Tucuruí 68455-901, Pará, Brazil

**Keywords:** stability, gene expression normalization, RT-qPCR

## Abstract

**Background/Objectives**: The selection and validation of species-specific housekeeping genes (HKGs) have become increasingly common in functional genomics, with application of quantitative Polymerase Chain Reaction (qPCR) or cDNA-based qPCR (RT-qPCR). Despite the *Macrobrachium amazonicum* having RNA-seq studies available, there are still no data on the most stable and consistent HKGs for use in relative gene expression analyses. Therefore, the present study aimed to identify and validate seven HKGs in *M. amazonicum*: Eukaryotic Translation Initiation Factor (EIF), 18S ribosomal RNA (18S), Ribosomal Protein L18 (RPL18), β-actin, α-tubulin (α-tub), Elongation Factor 1-α (EF-1α), and Glyceraldehyde-3-phosphate Dehydrogenase (GAPDH). **Methods**: The HKGs were identified in the *M. amazonicum* transcriptome, characterized for identity confirmation, and compared against public databases. Subsequently, RT-qPCR assays were prepared using muscle, hepatopancreas, gills, testis, androgenic gland, and ovary to assess the stability of the HKG markers, employing the comparative ∆Ct, BestKeeper, NormFinder, and GeNorm methods. **Results**: All candidate HKGs identified showed high similarity with other decapods. Reactions performed with these markers demonstrated high specificity, PCR efficiency, and elevated coefficients of determination. The comprehensive ranking, indicated that no single HKG was stable across all tissues, with HKGs showing the best stability being tissue-specific. The most stable HKGs were RPL18 and 18S. GAPDH, historically used as an HKG, showed the poorest performance in stability ranking for most tissues tested, whereas β-actin was most suitable only for ovarian. **Conclusions**: These data reinforce the need for species-specific HKG validation and provide an appropriate panel of reference markers for gene expression studies in the *M. amazonicum*.

## 1. Introduction

Real-time Polymerase Chain Reaction (qPCR) is a highly sensitive and specific technique for detecting and quantifying nucleic acids, capable of identifying fewer than five copies of a target sequence [1,2]. By measuring fluorescence emitted by intercalating dyes or gene-specific probes, qPCR enables not only amplification but also quantification of DNA or cDNA in the RT-qPCR variant [3,4]. Owing to its reproducibility, low cost, and broad applicability, it has become a key tool in gene expression analyses, including those involving non-model organisms [5,6,7,8].

Accurate qPCR normalization requires the use of reference or housekeeping genes (HKGs), whose stable expression allows reliable comparison of target transcript levels. These genes are typically associated with essential cellular functions such as protein synthesis and energy metabolism [9], and should remain consistently expressed regardless of tissue type or physiological condition [10,11]. Although β-actin and glyceraldehyde-3-phosphate dehydrogenase (GAPDH) have historically been employed as universal HKGs in vertebrates and invertebrates [11,12], accumulating evidence indicates that no gene exhibits uniform stability across taxa, reinforcing the need for species-specific validation [13,14,15]. In this effort, online resources such as Internal Control Genes (ICG) [16] and RGeasy [17] provide curated catalogs of potential reference markers for different organisms.

Housekeeping gene validation has become especially relevant for decapod crustaceans, where RNA-seq approaches have advanced our understanding of nutrition, growth, reproduction and immune response [18,19,20,21,22]. In several species of crabs, crayfish, shrimp and prawns, including *Macrobrachium*, ribosomal proteins (RPLs), elongation and translation factors (EF1-α, EIF), and 18S rRNA have shown greater stability than classical β-actin or GAPDH [7,23,24,25], highlighting the need for prior experimental validation to ensure accurate expression profiling.

The Amazon River prawn *Macrobrachium amazonicum* is widely distributed throughout South American river basins [26,27] and represents one of the most biologically and economically relevant freshwater decapods in Brazil, ranking as the third most studied species of the genus [28]. Transcriptomic studies have expanded knowledge on nutrition [29,30,31] and immune pathways [32], yet no validated reference genes have been established for RT-qPCR normalization. Consequently, many studies continue to rely on non-validated classical genes, which may introduce normalization bias and compromise the reproducibility and biological interpretation of expression data. This gap represents a methodological bottleneck for molecular research involving *M. amazonicum*.

In this context, our objective was to identify and validate candidate housekeeping genes in *M. amazonicum* using transcriptome-derived sequences, selecting those with the highest expression stability across commonly studied tissues. We hypothesized that ribosomal and translation-related genes (RPL18, EIF, EF1-α, 18S rRNA) would display more stable expression than glycolytic (GAPDH) or cytoskeletal markers (β-actin, α-tubulin), and that no single gene would be universally suitable across tissues. We further evaluated expression uniformity between sexes to ensure the selection of robust and broadly applicable reference markers for future gene expression studies in this species.

## 2. Materials and Methods

### 2.1. Sampling

The dataset used in this study was derived from a cDNA library constructed using hepatopancreas tissue from a pool of ten adult males of *M. amazonicum*, approximately four months old. Specimens were collected from earthen pond nurseries at the Aquaculture Center of UNESP (CAUNESP), Jaboticabal, São Paulo, Brazil. These individuals are descendants of a native population from the estuary of Mosqueiro Island, located in the Amazon coastal region of Pará State, northern Brazil (01°12′37.7″ S, 46°08′17.1″ W).

### 2.2. Total RNA Extraction, Library Preparation, and Sequencing

The animals were initially anesthetized in water at 4 °C, followed by tissue collection and storage in RNAlater (Sigma-Aldrich, St. Louis, MO, USA). Total RNA was extracted using the PureLink^®^ RNA Mini Kit (Life Technologies, Carlsbad, CA, USA), according to the manufacturer’s instructions. Total RNA was treated with TURBO™ DNase prior to reverse transcription, following the manufacturer’s recommendations. Complementary DNA (cDNA) was then synthesized using the High-Capacity cDNA Reverse Transcription Kit (Applied Biosystems, Foster City, CA, USA). The integrity and quality of the material were assessed by 1.5% agarose gel electrophoresis and quantified using a NanoDrop Lite Plus spectrophotometer. The cDNA library was prepared with the TruSeq^®^ RNA LT Sample Preparation Kit v2, and sequencing was performed on the Illumina HiSeq 2500 platform (Illumina, San Diego, CA, USA) using the TruSeq SBS v3-HS kit (Illumina, San Diego, CA, USA).

### 2.3. Bioinformatics Analyses and Identification of Housekeeping Genes (HKGs)

After sequencing, low-quality reads (Q < 20) were identified and removed using FastQC 0.12.0 [33] and Trimmomatic 0.39 [34]. The cleaned reads were then assembled de novo, without a reference genome, using Trinity 2.15.2 [35]. Candidate housekeeping genes (HKGs) were identified within the database generated from the transcriptome assembly using MEB 0.9.2 [36], through the implementation of the local BLASTn algorithm. To optimize the searches, nucleotide sequences of the following genes were used as references: EIF, 18S, Ribosomal Protein L18 (RPL18), β-actin, α-tubulin (α-tub), EF-1α and Glyceraldehyde-3-phosphate Dehydrogenase (GAPDH), available for *Macrobrachium* species in NCBI, under the accession numbers: MH540106.1, AY461599.1, MH540112.1, AF221096.1, MH540110.1, KF228019.1, KF305552.1, respectively.

The sequences identified with MEB were visualized with BioEdit 7.1 [37]. Open reading frames (ORFs) were predicted in ORFfinder (https://www.ncbi.nlm.nih.gov/orffinder/ (accessed on 21 August 2025)), except for 18S, as it corresponds to a ribosomal RNA region. For the remaining genes, the predicted amino acid (aa) sequences were used to identify conserved domains using the Simple Modular Architecture Research Tool (SMART) [38]. The three-dimensional protein structures were also predicted based on homologous crystal structure models available in the Protein Data Bank (PDB) through Swiss-Model [39]. Secondary structures were defined using ENDscript 2.0 [40], and structural conformations were further edited and visualized using PyMOL 2.5.7 [41].

### 2.4. Multiple Alignments and Cladograms

In parallel, BLASTn searches were performed for each sequence to confirm gene identity, using the National Center for Biotechnology Information (NCBI) database as a reference (accessed on 20 August 2025). New datasets were constructed containing the *M. amazonicum* sequences alongside homologous sequences from closely related species available in NCBI. The retrieved sequences were automatically aligned using Clustal Omega 2.10.0 [42], after which conserved and semi-conserved regions were annotated with ESPript 3.0 [40].

Cladograms were constructed in IQ-TREE 1.6.12 [43] to depict the relationships between the candidate housekeeping genes (HKGs) identified in *M. amazonicum* and those of other decapod species available in the NCBI database. Phylogenetic trees were generated using the Maximum Likelihood (ML) method based on 1000 bootstrap pseudoreplicates. The evolutionary models applied were as follows: HKY + F + G4 for EIF, TIM2 + F + R3 for 18S, TIM2e + G4 for RPL18, TIM2e + I + G4 for β-actin, α-tub, EF-1α, and GAPDH.

### 2.5. Validation of Candidate Housekeeping Gene (HKG) Markers

For the validation of candidate markers, adult *M. amazonicum* specimens were collected from northeastern Pará, Brazil (Bragança, Pará, Brazil; 1°01′49.04″ S, 46°45′14.26″ W). Tissues were sampled in biological triplicates, including muscle (Mu), hepatopancreas (Hp), and gills (Gi), from both males (♂) and females (♀), sexed based on external morphology [44]. Additionally, testis (Te) and the androgenic gland (Ag) were sampled from males, and ovaries (Ov) from females. Total RNA isolation, cDNA synthesis, and assessment of RNA integrity and quality were performed as described in Section 2.2.

The primers for each candidate gene (Table 1) were designed using Primer Express 3.0, using the mRNA sequences non-exon spanning identified in the transcriptome with the following parameters: primer length (20–24 nt), amplicon size (90–250 nt), GC content (35–60%), and annealing temperature (59–61 °C). RT-qPCR assays were performed in a final volume of 10 µL, consisting of 0.4 µL of each primer at 10 µM, 5 µL of PowerUp™ SYBR™ Green Master Mix (Thermo Fisher, Waltham, MA, USA), 1 µL of cDNA, and 3.2 µL of ultrapure water to complete the final volume. No-template controls (NTCs) were included to verify the absence of contamination. Each reaction was run in technical duplicates to assess the consistency of amplification. In summary, the reactions were conducted with three biological replicates x two technical replicates.

The reactions were run on a StepOnePlus™ Real-Time PCR System, with the following cycling conditions: 95 °C for 20 s, followed by 40 cycles of 95 °C for 3 s and 60 °C for 30 s. A final dissociation step was included: 95 °C for 15 s, 60 °C for 1 min, and 95 °C for 15 s.

### 2.6. Marker Specificity and Amplification Efficiency

The specificity of the candidate HKG markers was assessed through agarose gel electrophoresis, amplicon size, and melting curve analysis. Amplification efficiency was evaluated using a linear regression model and calculated from the slope of a standard curve. Efficiency (E) and the correlation coefficient (R^2^) were determined for each HKG using R v4.4.2 [45].

For the evaluation of both parameters, a pooled cDNA sample was prepared by combining all target samples (different tissues × sexes × biological triplicates) was prepared, using a serial dilution of the pooled samples (1:1, 1:4, 1:16, 1:64, 1:256), except for the β-actin and GAPDH genes, for which the undiluted point (1:1) did not yield an appropriate regression. An additional dilution point was therefore included (1:4, 1:16, 1:64, 1:256, 1:1024). For the construction of standard curves and calculation of E, dilution factors were plotted on a logarithmic scale (100, 10, 1, 0.1, and 0.01).

### 2.7. Methods for Analyzing HKG Stability

To assess the stability of the HKGs, RT-qPCR assays were performed on all individual samples (different tissues × sexes × biological triplicates). The resulting cycle threshold (Ct) values were used to evaluate gene expression stability across the different samples using RefFinder [46], which integrates four widely used computational algorithms for stability testing: comparative ∆Ct [47], BestKeeper [48], NormFinder [49], and GeNorm [50], generating a comprehensive ranking that considers all four methods.

Ct values were also analyzed to assess potential differences in gene expression among tissues and between sexes using R 4.4.1 [45]. Normality and homogeneity of variance were tested using Shapiro–Wilk and Levene’s tests, respectively. As the assumptions of normality and homoscedasticity were not met, non-parametric statistical tests were applied. The Kruskal–Wallis test was used with a significance level of 5%, followed by Dunn’s post hoc test to identify specific differences between gene × sex combinations. Graphs were generated using the ggplot2 package [51].

## 3. Results

### 3.1. Gene Identification and Characterization

**Figure 1:**
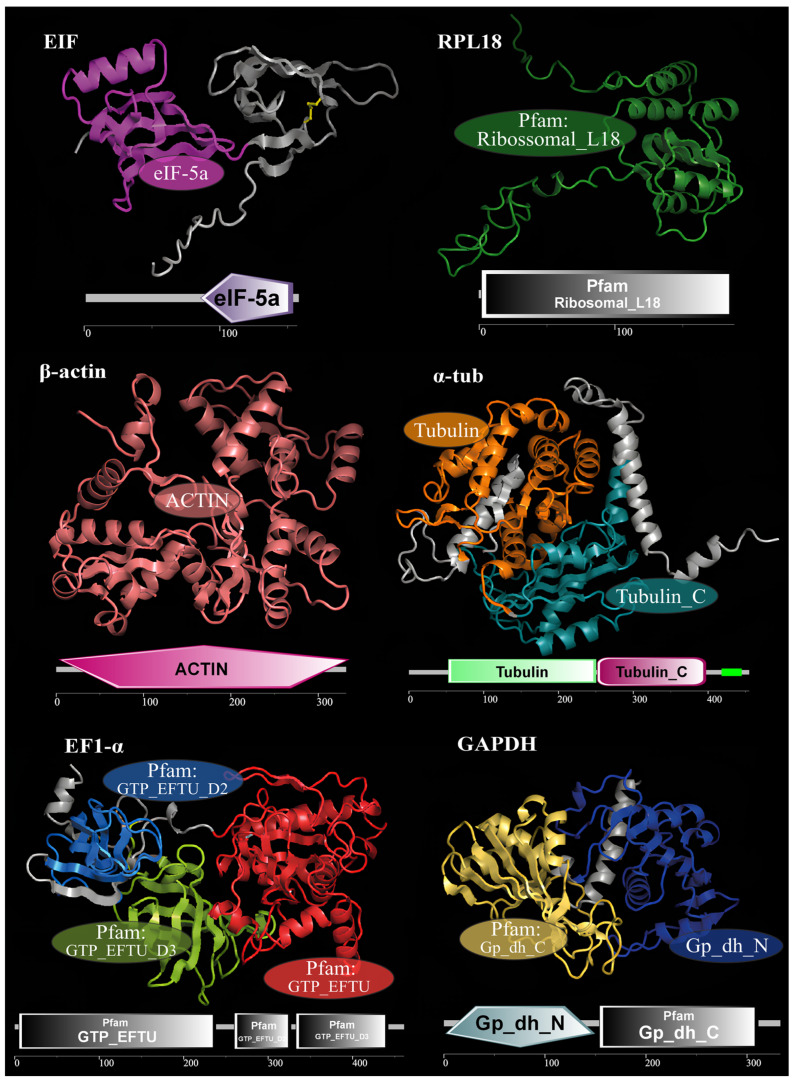
Housekeeping gene (HKG) candidates detected in the *Macrobrachium amazonicum* transcriptome, with the conserved domains of each gene highlighted in the spatial conformation of the proteins, together with the representation of the one-dimensional linear sequence. The 18S gene was not represented because no homologous three-dimensional structure could be modeled.

The search of the *M. amazonicum* hepatopancreas transcriptome led to the identification of seven candidate HKGs (accession numbers: PX278678.1–PX278683.1, PX279125.1), all featuring complete coding sequences, including start and stop codons. Sequence lengths ranged from 630 to 2621 nucleotides for RPL18 and EIF, respectively (Table 2). Except for 18S, which corresponds to ribosomal RNA, all genes exhibited conserved domains and homologous crystal structures (Figure 1). The predicted protein models showed high structural similarity to the registered crystal structures, with identity values ranging from 68.6 to 98.8% (Table S1).

**Figure 2:**
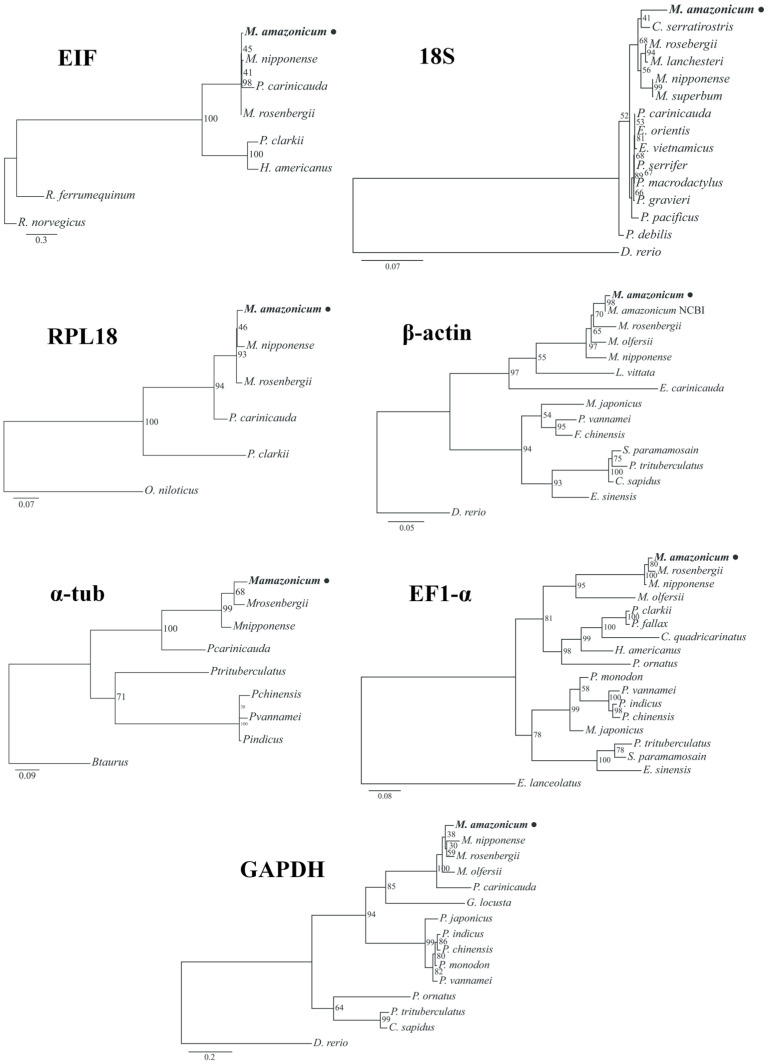
Maximum Likelihood cladograms based on 1000 bootstrap pseudoreplicates showing the phylogenetic relationships of the seven HKG candidates from *Macrobrachium amazonicum* and other decapod crustaceans retrieved from NCBI. All genes clustered within the Palemonidae lineage with high bootstrap support.

BLASTn analyses confirmed the identity of the HKGs, with the retrieved sequences showing high nucleotide similarity to orthologous genes from other decapod crustaceans, particularly species within the *Macrobrachium* genus. The highest similarity values were observed for β-actin (99.5%) and the lowest for GAPDH (89.1%) in *M. amazonicum*. Comparisons with other crustacean taxa revealed lower similarity values, with GAPDH and RPL18 showing 80.5% and 74.0% identity to *Callinectes sapidus* and *Procambarus clarkii*, respectively (Table 2; Figure S1A–G). The constructed cladograms depicted the phylogenetic relationships of *M. amazonicum* genes relative to other decapod sequences in NCBI, showing strong branch support within the Palaemonidae, and grouping vertebrate organisms as outgroups (Figure 2).

### 3.2. Specificity and Efficiency of Housekeeping Markers

**Figure 3:**
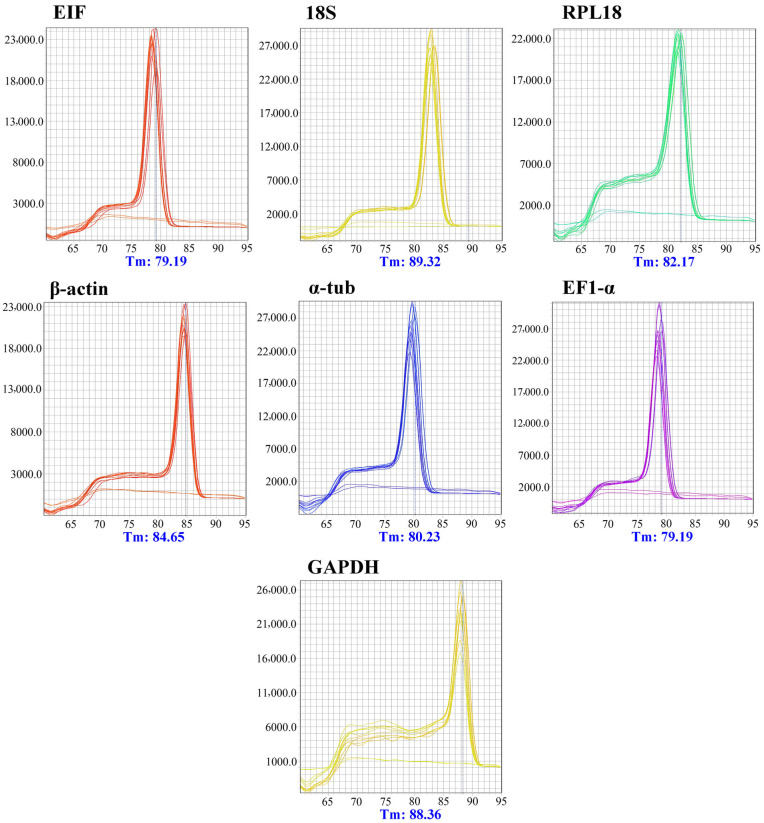
Melting curve profiles showing the specificity of the seven HKG candidate markers evaluated in pooled *Macrobrachium amazonicum* samples.

**Figure 4:**
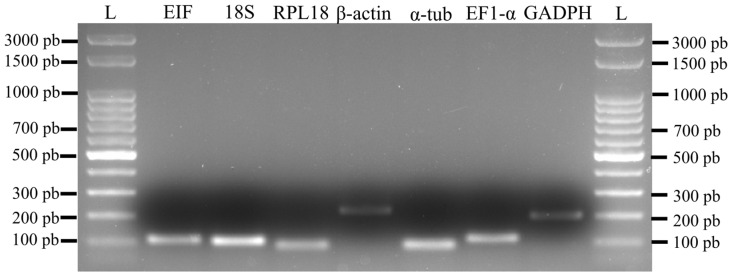
Amplification of the seven HKG candidate genes in pooled *Macrobrachium amazonicum* tissues, showing the specificity of the markers. Amplicon sizes ranged from approximately 100 to 250 nt. A 100 nt DNA ladder (L) was used as a size reference.

**Figure 5:**
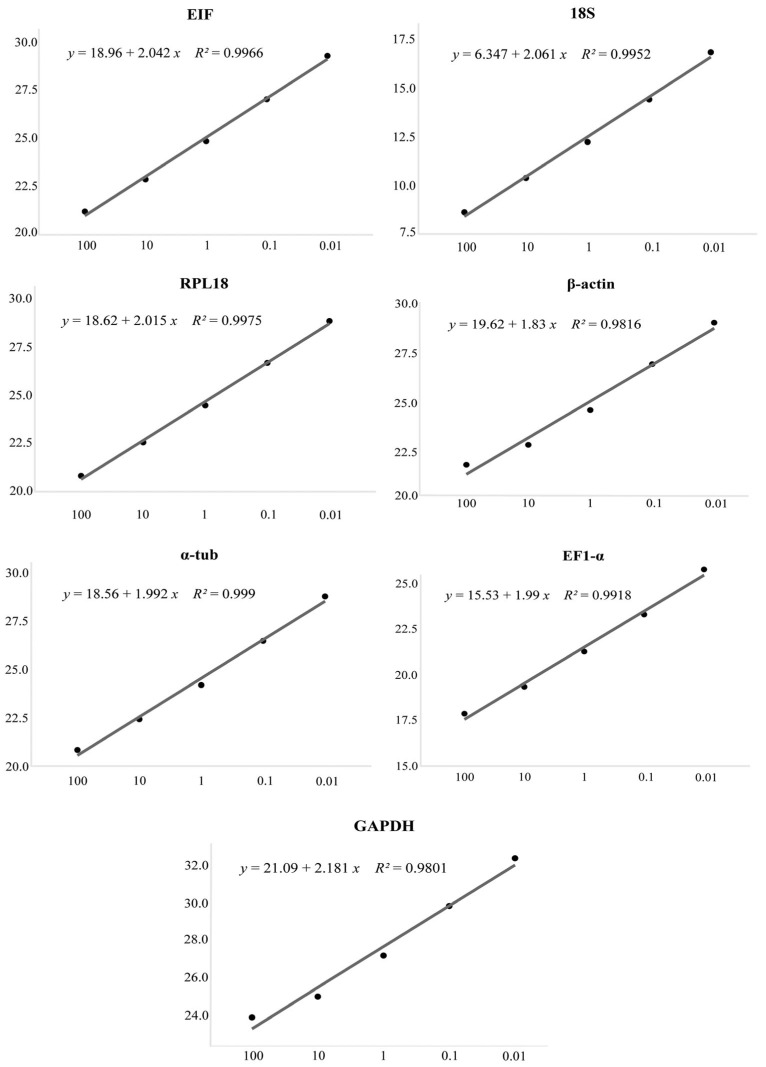
Dissociation curves and standard curves for the seven reference genes (HKGs) identified in *Macrobrachium amazonicum*. The standard curves were generated from a serial dilution of pooled cDNA (100, 10, 1, 0.1, 0.01) and are presented by plotting Ct values against the logarithm of template concentration. The regression equations displayed in each plot correspond to the calibration line used to calculate amplification efficiency, reflecting the linearity and performance of each gene in qPCR assays.

Amplifications initially performed using the pooled sample (different tissues × sexes × biological triplicates) confirmed the specificity of the candidate markers through melting curve analysis, which showed no primer-dimer formation or non-specific amplification products (Figure 3). Additional confirmation of HKG specificity was provided by agarose gel electrophoresis, which revealed a single band for each gene corresponding to the expected amplicon size (Figure 4). Reaction efficiencies ranged from 92.1% to 100.6%, and the standard curves generated from serial dilutions exhibited coefficients of determination (R^2^) greater than 0.98 (Table 1; Figure 5).

**Figure 6:**
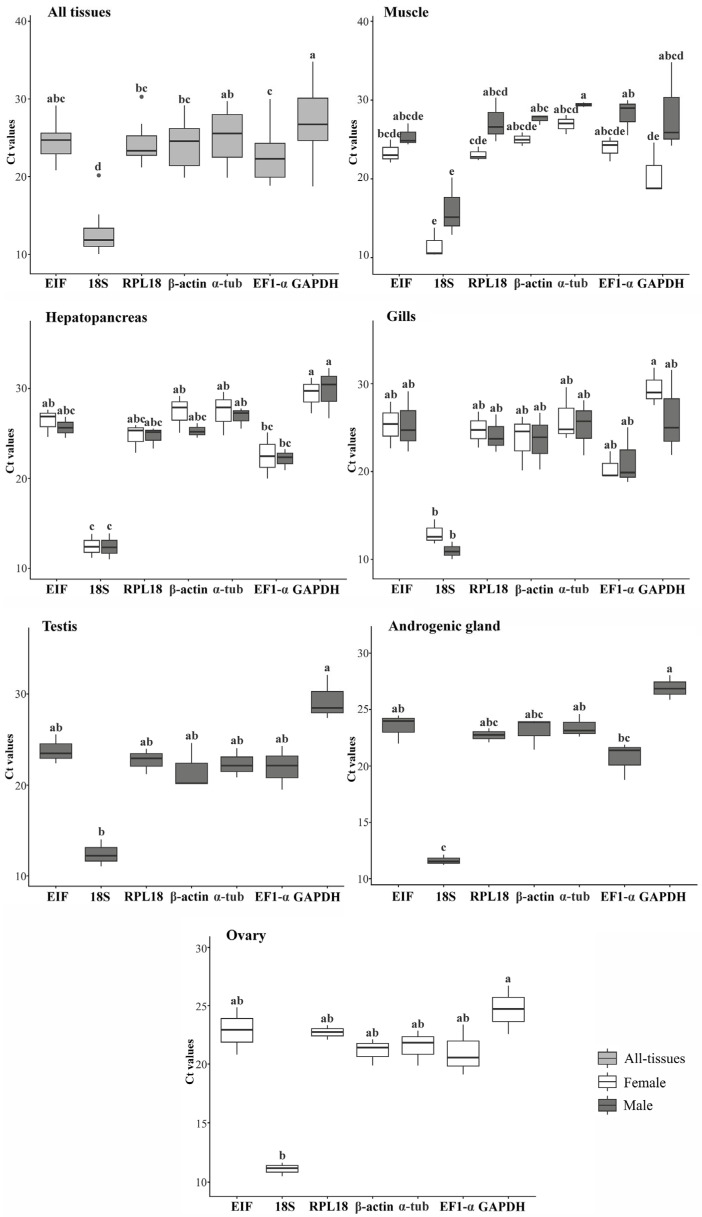
Expression levels of the HKG candidates in *Macrobrachium amazonicum* across different tissues. Cycle threshold (Ct) values were compared considering all tissues combined and individually between males and females, where applicable. Statistical tests were selected according to data normalit. Variables with the same letter indicate no statistically significant differences between means. Variables with different letters are significantly different (Kruskal–Wallis test; *p* < 0.05).

**Figure 7:**
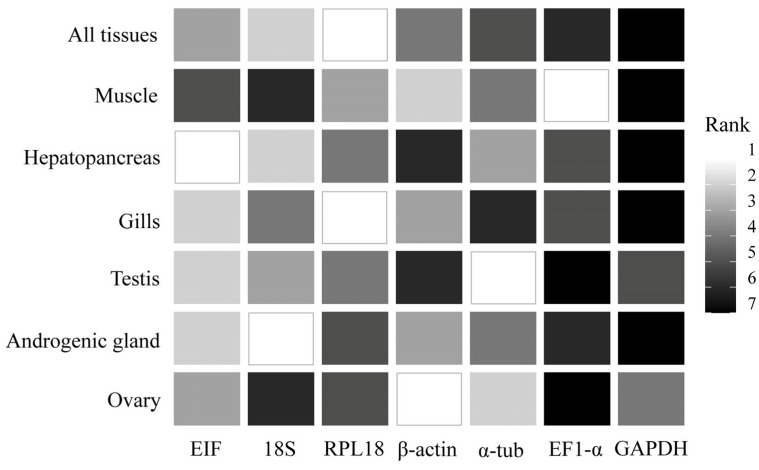
Summary of the comprehensive ranking of the most stable tissue-specific HKGs in *Macrobrachium amazonicum*, obtained using the four algorithms implemented in Reffinder: comparative ∆Ct, BestKeeper, NormFinder and geNorm.

Cycle threshold (Ct) values obtained from individual samples indicated that 18S was the most abundant transcript, consistently exhibiting the lowest Ct values. Expression of 18S differed significantly from the other tested HKGs (Kruskal–Wallis test; *p* < 0.05), which showed comparatively lower abundance across tissues of both sexes (Figure 6). Comparison of gene expression between sexes revealed greater variation in muscle tissue for most genes, while in gill tissue, variation was also observed for 18S and GAPDH. In the testis and the androgenic gland, both 18S and GAPDH exhibited significantly different expression levels between sexes (*p* < 0.05) (Figure 7).

### 3.3. Stability of Candidate HKGs

Stability tests using traditionally applied methods, such as comparative ∆Ct, BestKeeper, NormFinder, and GeNorm, identified different HKGs as the most stable across the various tissues of *M. amazonicum*. The BestKeeper method indicated 18S as the most stable gene in five tissue types (all tissues, hepatopancreas, gills, androgenic gland, and ovary). The other methods did not consistently identify a single HKG across tissues. The comparative ∆Ct method ranked α-tub as the most stable, but only in the hepatopancreas, testis, and ovary. NormFinder identified RPL18 as the most stable in three tissue types: all tissues, muscle, and gills. GeNorm reported two HKGs with the highest stability scores for each tissue analyzed (Table S2).

The comprehensive ranking generated by RefFinder, which integrates all four methods, identified RPL18 as the most stable HKG in two cases: all tissues and gills. For the remaining tissues, different HKGs were ranked as the most suitable. Notably, β-actin was identified as the most stable gene in the ovary, while GAPDH consistently ranked as the least stable across most tissues where it was evaluated, including all tissues, muscle, hepatopancreas, gills, and the androgenic gland (Figure 7).

## 4. Discussion

Validation of reference genes is a fundamental requirement for reliable normalization in gene expression studies, particularly in RT-qPCR, where accuracy directly depends on stable internal controls [1]. In this study, we identified and validated seven candidate housekeeping genes (HKGs) for *Macrobrachium amazonicum*, establishing a tissue-specific reference framework that supports gene expression quantification in this non-model crustacean.

The expansion of transcriptome-derived datasets has facilitated the discovery of novel candidate HKGs, allowing selection based on empirical stability rather than historical convention [6,69,70]. In *M. amazonicum*, all seven genes identified from hepatopancreas transcriptome sequences were supported by conserved protein domains, high nucleotide identity, and structural consistency, and clustered phylogenetically with orthologs from related *Macrobrachium* species [23,24,56]. This evolutionary conservation reinforces their roles in essential cellular processes, cytoskeletal organization, translational machinery and energy metabolism, which generally require stable expression across tissues and developmental states [71].

Mitochondrial markers such as COI have been used as positive controls in conventional RT-PCR in *M. amazonicum* [29,30], yet they are not recommended for gene expression normalization due to mitochondrial transcription being uncoupled from nuclear regulation. Variations in mitochondrial copy number, energy demand, or oxidative stress may strongly affect their expression [72,73,74], highlighting the need for validating nuclear-encoded HKGs to avoid normalization bias.

All RT-qPCR reactions in this study demonstrated adequate specificity and amplification efficiency, confirming the suitability of the sequences selected. However, stability analyses using ΔCt, BestKeeper, NormFinder, and GeNorm converged in showing that no single gene remained uniformly stable across all tissues. Instead, stability was tissue-dependent, a trend also observed in *M. rosenbergii* [24], *M. nipponense* [23], *P. monodon* [75] and *P. clarkii* [7]. Therefore, gene expression studies in decapods must adopt tissue-specific normalization strategies rather than relying on universal HKGs.

Among the candidates tested, 18S rRNA consistently showed high performance, ranking first in four tissues (hepatopancreas, gills, androgenic gland and ovary) and in pooled analyses. Although the comprehensive RefFinder index ranked 18S first only in the androgenic gland, its abundance and central role in ribosome assembly align with its stability [76]. Nonetheless, sex-dependent variation in muscle and gills indicates that 18S expression may be influenced by tissue- or hormone-dependent regulatory dynamics, an important consideration for sexually dimorphic analyses. Furthermore, 18S may often not be the best choice as HKG, due to its high expression, which is generally higher than the target genes in studies. Another aspect to consider is that rRNAs may exhibit biased stability due to differences compared to mRNAs, such as degradation and rRNA:mRNA ratio [11].

Including tissues from both sexes is crucial for studies focusing on sexual differentiation pathways, such as those involving IAG and related regulatory circuits [77,78]. In our results, GAPDH exhibited sex-specific differences in muscle and gills, similarly to observations in zebrafish [79], suggesting possible modulation by hormonal or metabolic factors.

For multi-tissue studies, genes that remain stable across sample types are preferred [71]. In this context, RPL18 and 18S emerged as the best general-use markers, presenting low variation in all-tissue analysis. It is noteworthy, however, that RPL18 is not universally stable: it ranked highly in adult *M. nipponense* but poorly during embryogenesis [23], emphasizing the importance of developmental validation before experimental application.

Conversely, GAPDH and β-actin, historically used in normalization [11], were not among the most stable genes in this study. GAPDH showed the lowest stability values across most tissues, matching patterns reported in multiple crustaceans [6,7,23,75]. In mammals, GAPDH is additionally influenced by microRNA regulation [15] and gene duplications [80], which can affect quantification accuracy, reinforcing the risk of relying on classical markers without validation.

β-actin exhibited high stability only in ovarian tissue, but not consistently among other tissues. Its responsiveness to developmental, environmental and chemical stimuli [14,15,81] questions its suitability as a universal reference. Still, its stability has been demonstrated under specific contexts, including stress exposure or RNAi induction in *M. nipponense* [23], and pathogen challenge in *M. nipponense* [82] and *C. semilaevis* [83]. These results illustrate that β-actin remains a viable reference when validated under the appropriate biological scenario.

The tissue-specific panels generated herein constitute a foundation for investigating male morphotypes and sexually dimorphic traits in *M. amazonicum*. By enabling accurate normalization in tissues such as testis, muscle and androgenic gland, these data support analyses of IAG signaling pathways, spermatogenic dynamics, developmental trajectories and transitions between morphotypes. Reduction of normalization bias is therefore expected to improve the resolution of comparative expression analyses across sexes, ontogenetic phases and social contexts, advancing mechanistic understanding and applied breeding strategies in aquaculture.

Our findings provide an empirically supported baseline for extending HKG validation in *M. amazonicum* to additional stages, including larval development [84], male morphotypes [85] and pathogen-induced stress. Expanding validation in these contexts will refine the robustness of reference markers across experimental conditions. As the use of non-validated HKGs can distort biological interpretation and compromise reproducibility [86], the present dataset represents a significant methodological step forward for gene expression research in this species. The limitation of this study is that the designed primers are not exon-spanning, therefore it has to be ensured that DNA contamination is minimized in the RNA extraction protocol. Moreover, future genomic studies of Amazon River prawn could allow for the creation of novel primer sets with improved RNA specificity.

To provide a practical guideline for future RT-qPCR studies in *M. amazonicum*, we compiled a summary table that lists the most stable pairs of reference genes for each tissue, together with alternative candidates for more complex normalization strategies (Table S3).

## 5. Conclusions

We performed the first identification and validation of housekeeping genes for *M. amazonicum*, establishing a panel of tissue-specific reference genes for use in adult specimens. The validated markers cover key tissues commonly employed in functional genomics, such as muscle, hepatopancreas, and gonad tissues, thereby enabling the selection of appropriate internal controls for accurate RT-qPCR-based gene expression analyses. By making these data publicly available through online platforms, this work contributes a valuable technological resource to support the design, execution, and reproducibility of gene expression analyses via RT-qPCR, serving as a technological tool applicable to the Amazon River prawn.

## Figures and Tables

**Table 1:** List of primers used in this study for evaluation of housekeeping gene (HKG) candidates. The table shows the expected amplicon size, GC content, annealing temperature (Ta), PCR efficiency (E), and correlation coefficient (R2), obtained from the qPCR assays.

Gene	Primer Sequence (5′-3′)	Length (bp)	GC%	Ta (°C)	E (%)	R^2^
EIF	F: GAGACTTGGGCACAGAAATC	115	50	61	97.14	0.9966
	R: TACTTCATGTTTGGCTTAGTAGC		39.1	61		
18S	F: GATTAAGTCCCTGCCCTTTG	110	50	60	95.97	0.9952
	R: GCTGGAAGAAACCACTAGAC		50	60		
RPL18	F: TGTCCAAAATTAACAAGCCTC	93	38.1	59	98.96	0.9975
	R: CCACAACAACAAAGATTCGC		45	60		
β-actin	F: CACGAGACCACCTACAATTC	223	50	60	95.67	0.9816
	R: GAGAAGCCAAGATAGAACCG		50	60		
α-tub	F: CATTCCGATTGTGCCTTTATG	94	42.9	60	100.57	0.9929
	R: TCAGGTTGGTGTATGATGGA		41	61		
EF1-α	F: TGTACCCATCATTCCCATTTC	120	42.9	60	100.68	0.9918
	R: GTCTCGTATTCATAAGATCCACTC		41.7	60		
GAPDH	F: TCCAGGTCTTCAACGAAATG	200	45	60	92.14	0.9801
	R: GTACTTCTCCAGGTTTACACC		47.6	60		

**Table 2:** Summary of HKG candidates identified in *Macrobrachium amazonicum*. The table presents the transcript and deduced amino acids (aa) lengths, nucleotide (nt) similarity with other decapods, accession numbers and references. Species used for multiple sequence alignments and for the reconstruction of gene-specific phylogenetic trees are also listed. * = *Macrobrachium amazonicum*.

Gene	Species	Nt	aa	% nt	Access NCBI	Reference
EIF	Macrobrachium amazonicum *	2621	157	-	PX278678.1	Present study
EIF	*Macrobrachium nipponense*	2872	210	95.5	MH540106.1	[23]
EIF	*Macrobrachium rosenbergii*	2291	157	96.9	XM_067081428.1	Unpublished
EIF	*Palaemon carinicauda*	2303	204	89.5	XM_068365025.1	Unpublished
EIF	*Procambarus clarkii*	1478	157	83.0	KR135170.1	[7]
EIF	*Homarus americanus*	3222	157	82.4	XM_042380784.1	Unpublished
EIF	*Rhinolophus ferrumequinum*	1573	153	80.9	XM_033122722.1	Unpublished
EIF	*Rattus norvegicus*	4871	153	79.7	XM_001063995.1	[52]
18S	Macrobrachium amazonicum *	2272	-	-	PX279125.1	Present study
18S	*Macrobrachium rosenbergii*	1844	-	96.2	DQ642856.1	Unpublished
18S	*Macrobrachium nipponense*	1902	-	96.5	XR_010313754.1	Unpublished
18S	*Palaemon carinicauda*	1902	-	96.4	XR_011045428.1	Unpublished
18S	*Macrobrachium superbum*	1885	-	96.4	KC515055.1	[53]
18S	*Palaemon gravieri*	1884	-	96.3	KC515058.1	[53]
18S	*Exopalaemon orientis*	1885	-	96.3	KC515053.1	[53]
18S	*Palaemon serrifer*	1884	-	96.3	KC515060.1	[53]
18S	*Caridina serratirostris*	1852	-	96.7	KP725709.1	[53]
18S	*Exopalaemon vietnamicus*	1884	-	96.1	KC515054.1	[53]
18S	*Palaemon pacificus*	1884	-	96.0	KC515059.1	[53]
18S	*Palaemon debilis*	1885	-	95.9	KC515057.1	[53]
18S	*Palaemon macrodactylus*	1855	-	96.3	DQ642849.1	Unpublished
18S	*Macrobrachium lanchesteri*	1852	-	96.2	KP725754.1	[54]
18S	*Danio rerio*	1887	-	82.5	XR_012407109.1	Unpublished
RPL18	Macrobrachium amazonicum *	630	188	-	PX278679.1	Present study
RPL18	*Macrobrachium nipponense*	672	188	96.5	MH540112.1	Unpublished
RPL18	*Macrobrachium rosenbergii*	710	188	96.7	XM_067089493.1	Unpublished
RPL18	*Palaemon carinicauda*	657	188	89.3	XM_068359494.1	Unpublished
RPL18	*Procambarus clarkii*	665	188	74.0	XM_045761550.2	Unpublished
RPL18	*Oreochromis niloticus*	649	188	82.0	NM_001279463.1	[55]
β-actin	Macrobrachium amazonicum *	1129	332	-	PX278680.1	Present study
β-actin	*Macrobrachium amazonicum* *	689	229	99.5	JX948081.1	Unpublished
β-actin	*Macrobrachium nipponense*	1324	376	97.1	KY780298.1	Unpublished
β-actin	*Macrobrachium olfersii*	1131	376	96.9	KY027067.1	[56]
β-actin	*Macrobrachium rosenbergii*	1281	376	96.4	AY626840.1	[57]
β-actin	*Exopalaemon carinicauda*	1335	376	91.1	JQ045354.1	Unpublished
β-actin	*Lysmata vittata*	1131	376	89.3	MT114194.1	Unpublished
β-actin	*Marsupenaeus japonicus*	1327	376	87.4	AB055975.1	Unpublished
β-actin	*Penaeus vannamei*	1249	376	87.1	MF627840.1	[58]
β-actin	*Fenneropenaeus chinensis*	1358	376	87.1	DQ205426.1	Unpublished
β-actin	*Scylla paramamosain*	1358	376	88.5	GU992421.1	Unpublished
β-actin	*Callinectes sapidus*	1338	376	88.1	DQ084066.1	[59]
β-actin	*Portunus trituberculatus*	1382	376	87.9	KC131030.1	Unpublished
β-actin	*Eriocheir sinensis*	1425	376	86.9	KY356885.1	Unpublished
β-actin	*Danio rerio*	1143	375	87.0	AF025305.1	[60]
α-tub	Macrobrachium amazonicum *	1700	455	-	PX278681.1	Present study
α-tub	*Macrobrachium rosenbergii*	2312	451	93.5	XM_067133707.1	Unpublished
α-tub	*Macrobrachium nipponense*	1172	356	92.5	MH540110.1	Unpublished
α-tub	*Palaemon carinicauda*	1664	451	86.4	XM_068357483.1	Unpublished
α-tub	*Portunus trituberculatus*	2106	450	83.9	XM_045281753.1	Unpublished
α-tub	*Penaeus chinensis*	1629	451	83.5	MW486011.1	[61]
α-tub	*Penaeus indicus*	1673	451	83.4	XM_063750237.1	Unpublished
α-tub	*Penaeus vannamei*	2211	451	83.3	XM_027367265.2	Unpublished
α-tub	*Bos taurus*	1921	451	82.7	NM_001166505.1	[62]
EF1-α	Macrobrachium amazonicum *	1795	461	-	PX278682.1	Present study
EF1-α	*Macrobrachium nipponense*	1762	461	97.9	XM_064243189.1	Unpublished
EF1-α	*Macrobrachium rosenbergii*	1386	461	97.7	OR130524.1	Unpublished
EF1-α	*Procambarus clarkii*	1673	461	83.1	XM_045749314.2	Unpublished
EF1-α	*Penaeus monodon*	1608	461	83.0	MG775229.1	Unpublished
EF1-α	*Procambarus fallax*	1568	461	82.7	LC035460.1	[63]
EF1-α	*Homarus americanus*	1633	461	82.5	XM_042379195.1	Unpublished
EF1-α	*Penaeus japonicus*	1550	461	83.1	AB458256.1	Unpublished
EF1-α	*Penaeus vannamei*	1658	461	82.8	XM_027373349.2	Unpublished
EF1-α	*Penaeus indicus*	1634	461	82.8	XM_063731077.1	Unpublished
EF1-α	*Penaeus chinensis*	1652	461	82.7	XM_047615957.1	Unpublished
EF1-α	*Cherax quadricarinatus*	1660	461	82.1	XM_070101441.1	Unpublished
EF1-α	*Panulirus ornatus*	1656	461	82.1	XM_071679762.1	Unpublished
EF1-α	*Portunus trituberculatus*	1633	461	82.6	KU361820.1	Unpublished
EF1-α	*Scylla paramamosain*	1559	461	81.9	JQ824130.1	[64]
EF1-α	*Macrobrachium olfersii*	1242	413	84.5	KY027069.1	[56]
EF1-α	*Eriocheir sinensis*	2050	461	80.9	KY356884.1	Unpublished
EF1-α	*Epinephelus lanceolatus*	1607	462	76.8	XM_033637922.1	Unpublished
GAPDH	Macrobrachium amazonicum *	1652	333	-	PX278683.1	Present study
GAPDH	*Macrobrachium nipponense*	1651	333	89.1	MH540109.1	Unpublished
GAPDH	*Macrobrachium rosenbergii*	1002	333	95.6	MH219928.1	Unpublished
GAPDH	*Macrobrachium olfersii*	1002	333	94.2	KY027066.1	[56]
GAPDH	*Palaemon carinicauda*	1514	333	86.3	KX893516.1	Unpublished
GAPDH	*Penaeus japonicus*	1826	449	86.9	XM_043022172.1	Unpublished
GAPDH	*Penaeus indicus*	1501	333	86.1	XM_063750341.1	Unpublished
GAPDH	*Penaeus monodon*	1711	414	85.7	XM_037920434.1	Unpublished
GAPDH	*Penaeus chinensis*	1761	430	85.7	XM_047617625.1	Unpublished
GAPDH	*Penaeus vannamei*	1492	332	86.0	MG787341.1	[65]
GAPDH	*Gammarus locusta*	1264	334	83.5	FM165079.1	Unpublished
GAPDH	*Panulirus ornatus*	1759	334	82.8	XM_071689634.1	Unpublished
GAPDH	*Portunus trituberculatus*	1457	334	80.9	EU919707.1	Unpublished
GAPDH	*Callinectes sapidus*	888	296	80.5	AAS02313.1	[66]
GAPDH	*Drosophila melanogaster*	2141	332	78.0	M11254.1	[67]
GAPDH	*Danio rerio*	1329	333	69.2	NM_001115114.1	[68]

## Data Availability

The data generated in this study has been deposited at the National Center for Biotechnology Information (NCBI) under the accession numbers PX278678.1–PX278683.1, PX279125.1. The sequences are currently private and will be made public with the publication of this study.

## Supplementary Materials

The following supporting information can be downloaded at: https://www.mdpi.com/article/10.3390/genes17010026/s1, Table S1. Homologous crystal structure models employed for the three-dimensional modeling of *Macrobrachium amazonicum* housekeeping gene (HKG) candidates [87,88,89,90,91]. The file reports the structural similarity (%) of the templates, the corresponding species, accession numbers, and the respective references, when available. * evaluation of amino acid (aa) similarity. ** specific taxon of the species not defined. Table S2. Summary of the stability tests for the HKG candidates in the different tissues of *Macrobrachium amazonicum*. Table S3. Recommended housekeeping genes for RT-qPCR normalization in *Macrobrachium amazonicum* based on integrated stability rankings obtained from comparative ΔCt, BestKeeper, NormFinder and geNorm algorithms, complemented by geNorm pairwise variation analysis. The ‘Primary recommended HKGs (pair)’ column lists the most stable pair of reference genes identified for each experimental context, whereas the ‘Alternative/additional genes’ column provides further candidates that can be combined when more than two reference genes are required or when specific gene classes (e.g., cytoskeletal genes) need to be avoided. The ‘Rationale’ column summarizes the main criteria used to select each combination of reference genes. Figure S1. Multiple alignments of the seven genes identified in *Macrobrachium amazonicum* with other decapod crustaceans available on NCBI (accessed on: 20 August 2025). Only regions in which the *M. amazonicum* sequence is covered are shown. The *consensus* > 70 shows the regions with more than 70% similarity recorded by the Clustal Omega algorithm.
