# Supplementary material for: Identification and Validation of Tissue-Specific Housekeeping Markers for the Amazon River Prawn Macrobrachium amazonicum (Heller, 1862)
Source: Genes (Basel). 2025 Dec 28;17(1):26. doi: 10.3390/genes17010026 (PMC12840830; doi:10.3390/genes17010026)
Supplement: Supplementary file 1 [file genes-17-00026-s001.zip › Table S3.pdf]

Table S3. Recommended housekeeping genes for RT-qPCR normalization in *Macrobrachium amazonicum* based on integrated stability rankings obtained from comparative ΔCt, BestKeeper, NormFinder and geNorm algorithms, complemented by geNorm pairwise variation analysis. The 'Primary recommended HKGs (pair)' column lists the most stable pair of reference genes identified for each experimental context, whereas the 'Alternative/additional genes' column provides further candidates that can be combined when more than two reference genes are required or when specific gene classes (e.g., cytoskeletal genes) need to be avoided. The 'Rationale' column summarizes the main criteria used to select each combination of reference genes.

**Experimental context**	**Primary recommended HKGs (pair)**	**Alternative/additional genes**	**Rationale**
All tissues (combined)	RPL18 + 18S	EIF	Top-ranked in the comprehensive RefFinder stability ranking and forming one of the most stable geNorm pairs across tissues; recommended for multi-tissue experimental designs
Muscle	EF1-α + β-actin	α-tub; RPL18	EF1-α and β-actin consistently appear among the top-ranked genes in muscle and show high expression stability, with α-tub and RPL18 as additional options when more than two reference genes are required
Hepatopancreas	EIF + 18S	α-tub; RPL18	EIF and 18S are systematically among the most stable genes according to comparative ΔCt, NormFinder and geNorm analyses in hepatopancreas, with α-tub and RPL18 as suitable complementary candidates
Gills	RPL18 + EIF	β-actin; 18S	RPL18 is the most stable gene in gills and EIF ranks among the top candidates, together forming a robust geNorm pair; β-actin and 18S can be used as additional alternatives if needed
Testis	α-tub + EIF	18S; RPL18	α-tub and EIF show the highest stability in testis and constitute the best-performing geNorm pair, whereas 18S and RPL18 can be considered as additional reference genes in more complex normalization strategies
Androgenic gland	18S + EIF	β-actin; α-tub	18S is the top-ranked gene in the androgenic gland and EIF is consistently placed among the most stable candidates, with β-actin and α-tub providing further options depending on experimental design
Ovary	β-actin + α-tub	EIF + GAPDH	β-actin and α-tub display the highest stability in ovary, whereas EIF + GAPDH can be used as an alternative pair in experiments where additional or non-cytoskeletal reference genes are desirable
