# Supplementary material for: Identification and Validation of Tissue-Specific Housekeeping Markers for the Amazon River Prawn Macrobrachium amazonicum (Heller, 1862)
Source: Genes (Basel). 2025 Dec 28;17(1):26. doi: 10.3390/genes17010026 (PMC12840830; doi:10.3390/genes17010026)
Supplement: Supplementary file 1 [file genes-17-00026-s001.zip › Figure S1.pdf]

Figure S1A–G. Multiple alignments of the seven genes identified in *Macrobrachium amazonicum* with other decapod crustaceans available on NCBI (accessed on: 20 August 2025). Only regions in which the *M. amazonicum* sequence is covered are shown. The *consensus>70* shows the regions with more than 70% similarity recorded by the Clustal Omega algorithm.

Figure S1A. EIF gene.

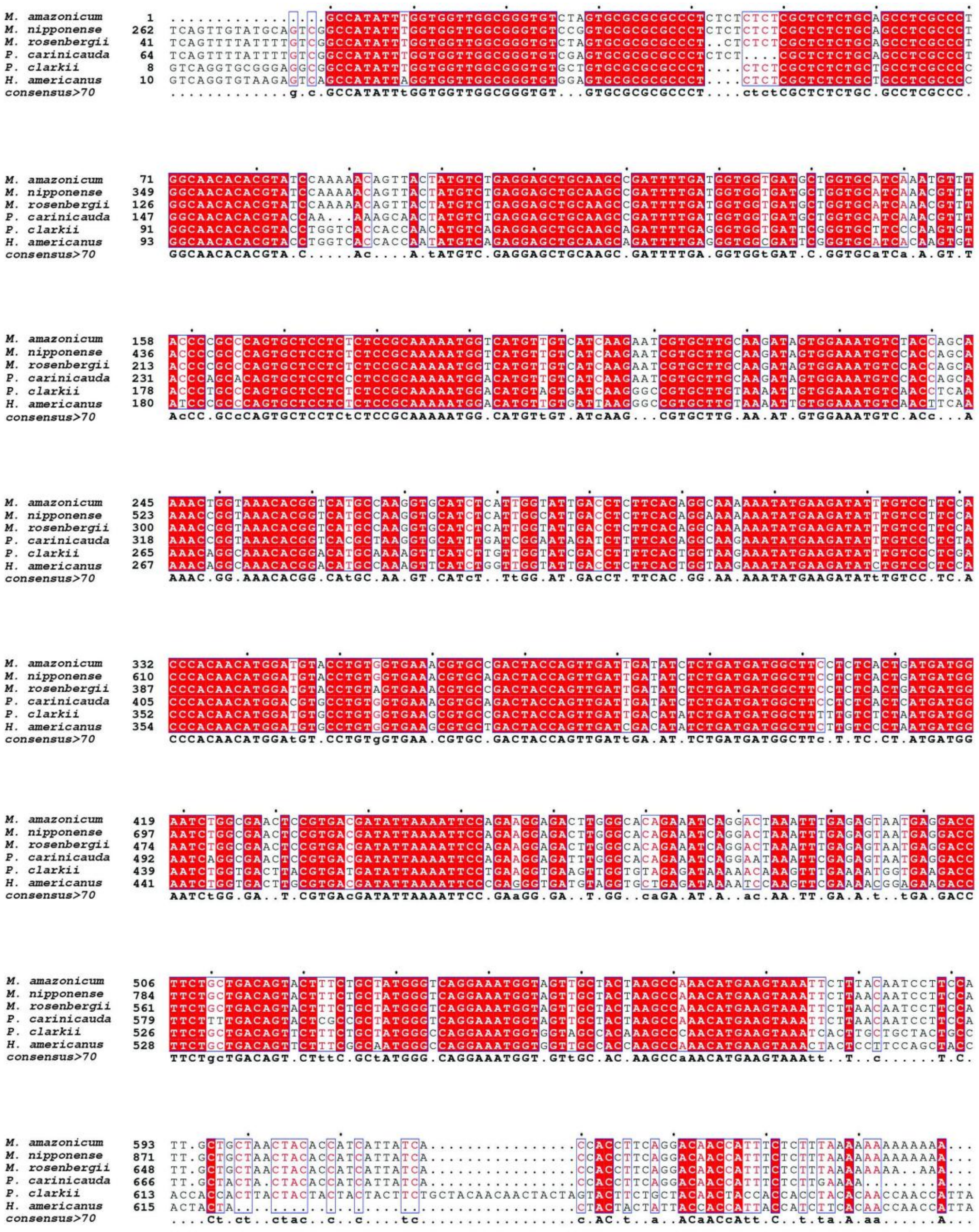

Figure S1A. EIF gene continued.

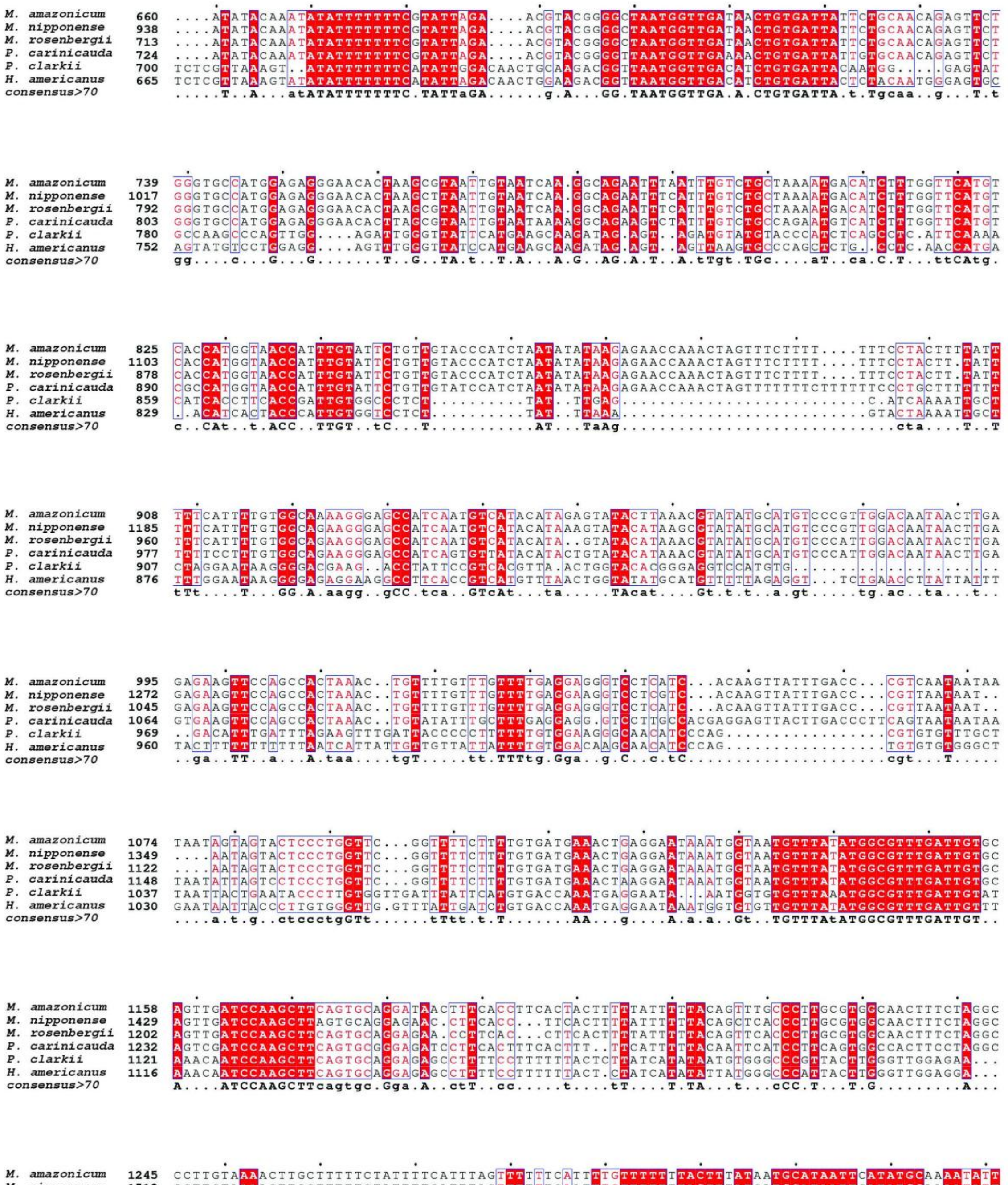

Figure S1A. EIF gene continued.

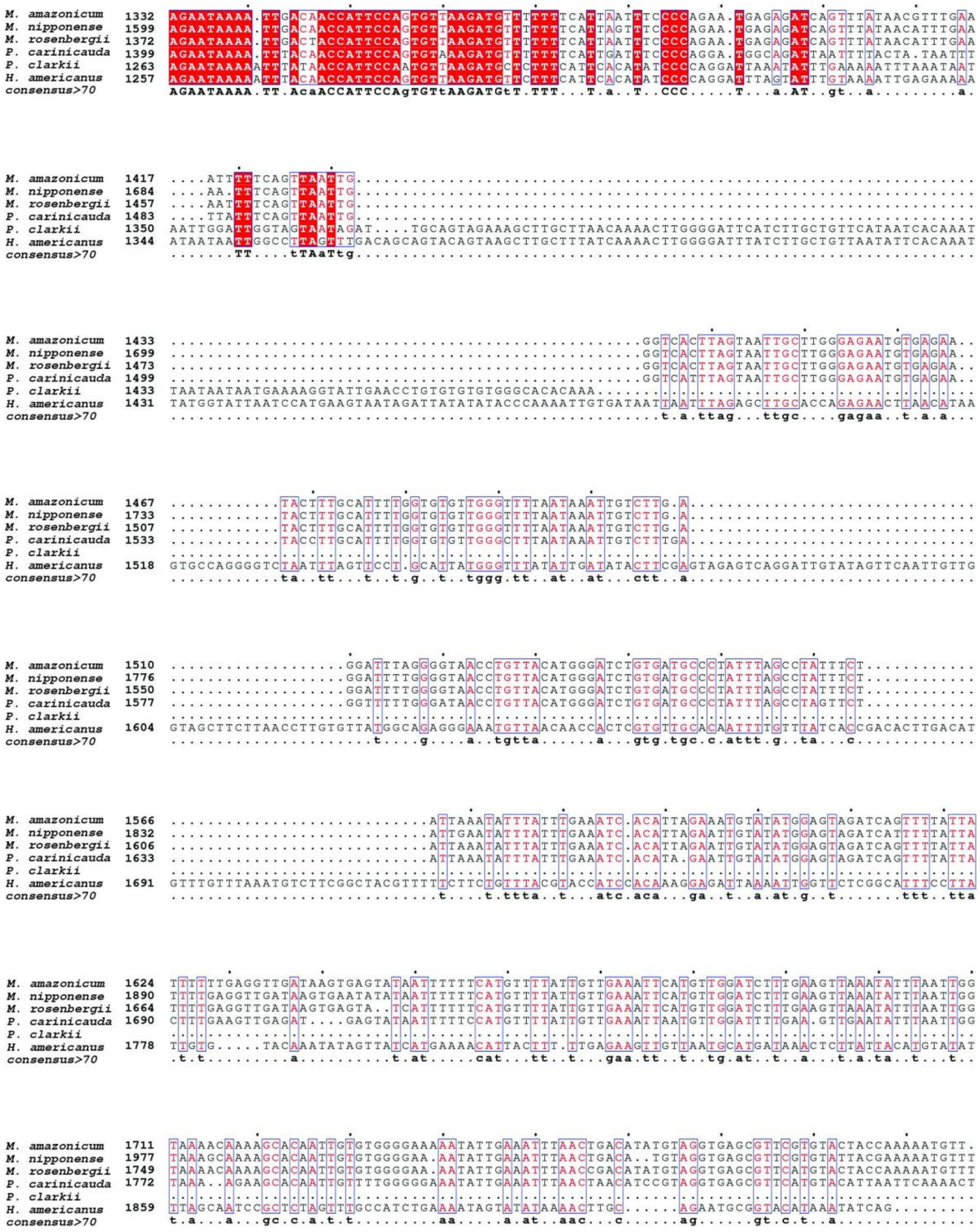

Figure S1A. EIF gene continued.

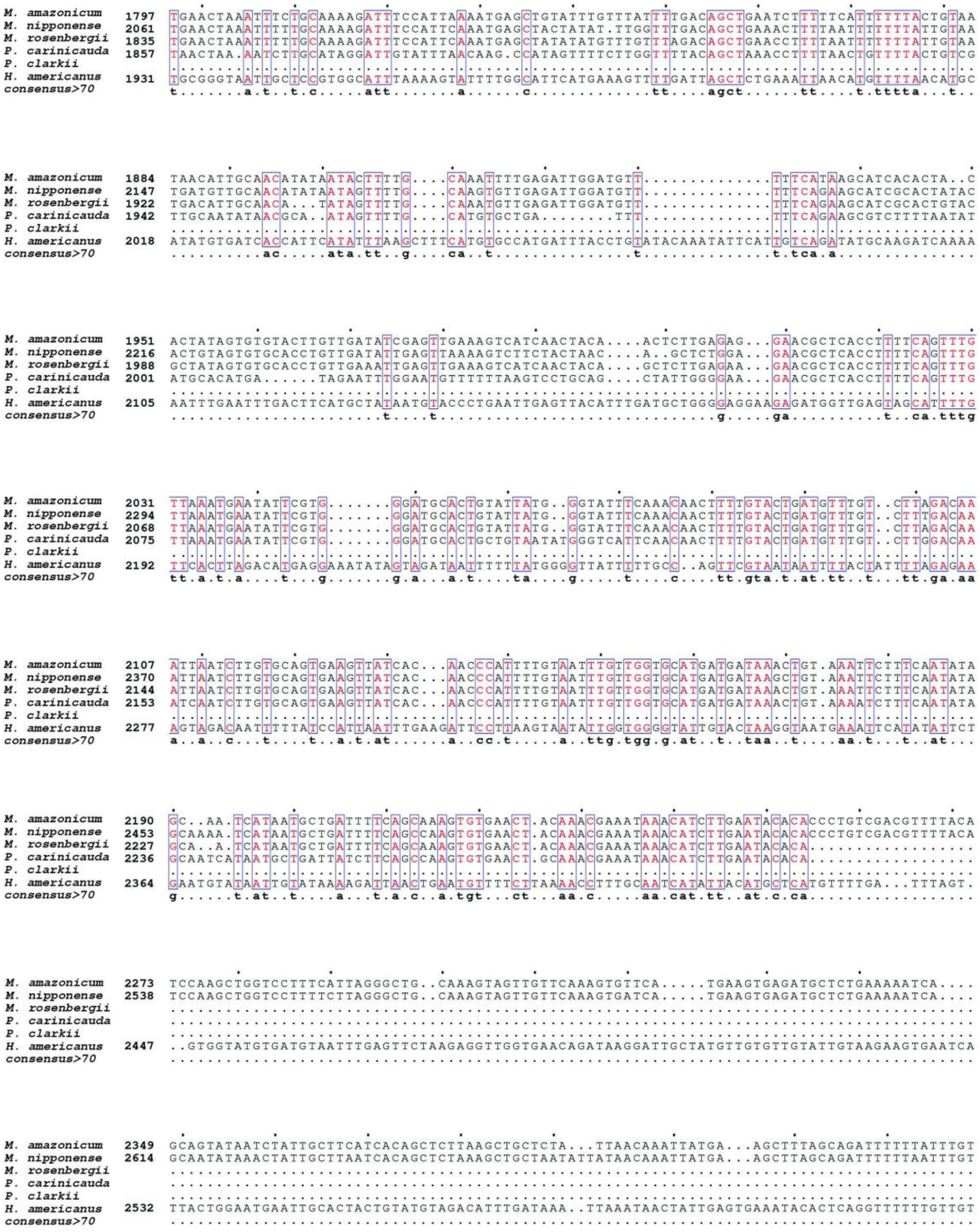

Figure S1A. EIF gene continued.

```
M. amazonicum   2430 CCACGGCTATCTCTTCAATGCTCAGGAATTTTGCAT..ATTCAGTTCGTTTGGCACTTATTTATTAGGCTATCGGCTTTTAAGGTA.
M. nipponense   2698 CCACGGCTATCTCTTCAATGCTCAGGAATTTTGCATATATTCAGTTCGTTTGGCACTTATTTATTAGGCTATCGGCTTTTAAGGTAT
M. rosenbergii       .......................................................................................
P. carinicauda       .......................................................................................
P. clarkii           .......................................................................................
H. americanus   2617 AGACTGACCCAAATGA...GAACAT.AGTGACTCAACGTATCAGTTCATTTGACAATTTAAATT.GCCATTTTTTATTCAAAGGTTT
consensus>70         .......................................................................................

M. amazonicum   2514 .............TTTGA.............CTTGCTTCATTTGCGTGAAATTTCCATGTATGTTG.....TACATGAGTATTGTAA
M. nipponense   2785 T............TTTGA.............CTTGCTTAATTTGCGTGAAATTTCCATGTATGTTG.....TACATGAGTATTGTAA
M. rosenbergii       .......................................................................................
P. carinicauda       .......................................................................................
P. clarkii           .......................................................................................
H. americanus   2699 ATATCCTCTGTACTTTGAGAAACTACAATTGCATACTAAGATTGCTTAAGCTTTAAAAACTTGCTGTTTTTCTCTGGAGTAAAGCAA
consensus>70         .......................................................................................

M. amazonicum   2570 AGTGTTTTATATATATATATATATATATATATATATA....TATATATATATATAT...............................
M. nipponense   2842 AGTGTTTTATATATATATATATAATATATAT........................................................
M. rosenbergii       .......................................................................................
P. carinicauda       .......................................................................................
P. clarkii           .......................................................................................
H. americanus   2786 AATGTTTTTATTTTCACAATGTAAATATTGACCTTCAAAATACAATAAATACATAATGGTTTTGTTTGTTAAATCTACTTGTAGATA
consensus>70         .......................................................................................
```

Figure S1B. 18S gene.

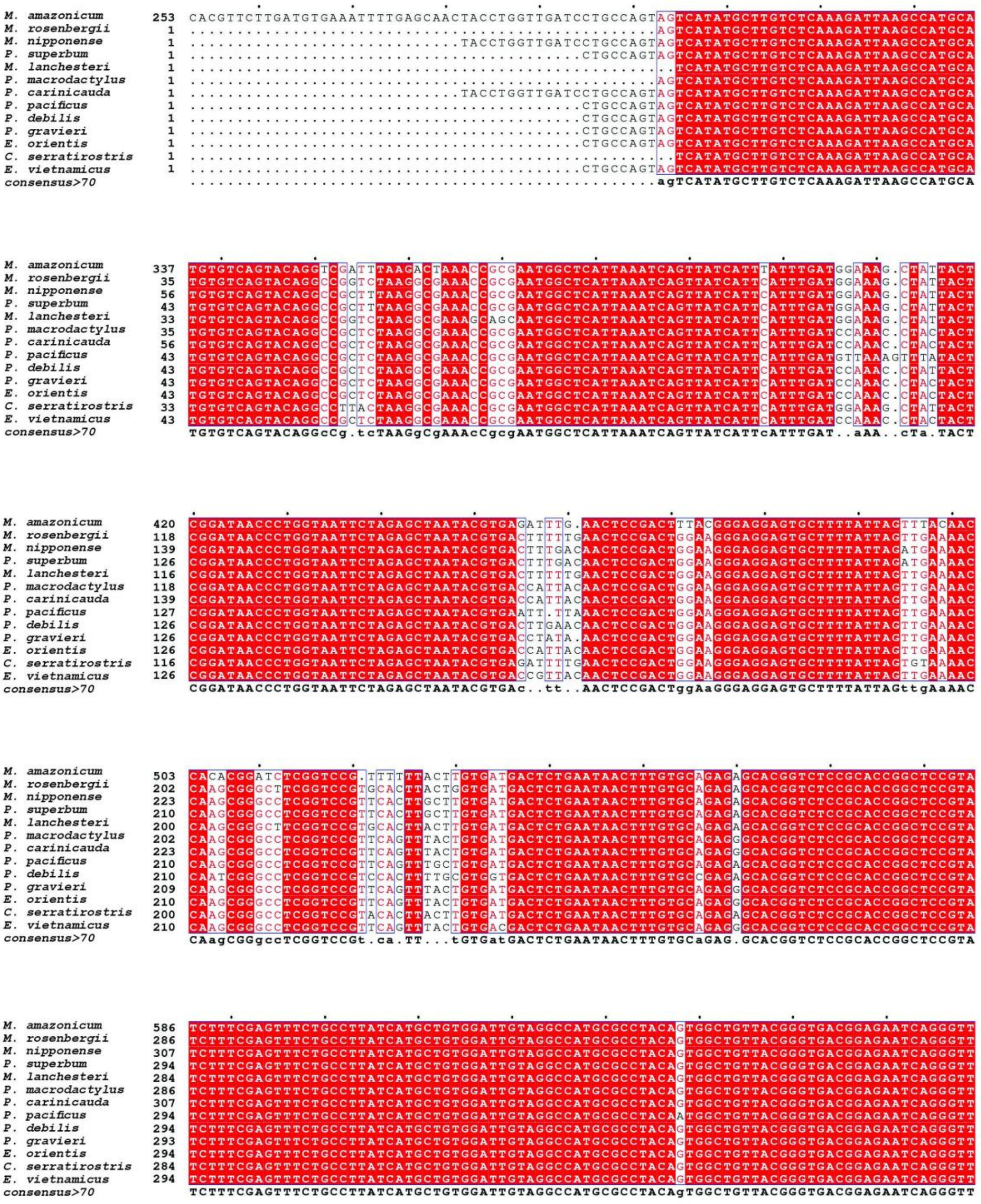

Figure S1B. 18S gene continued.

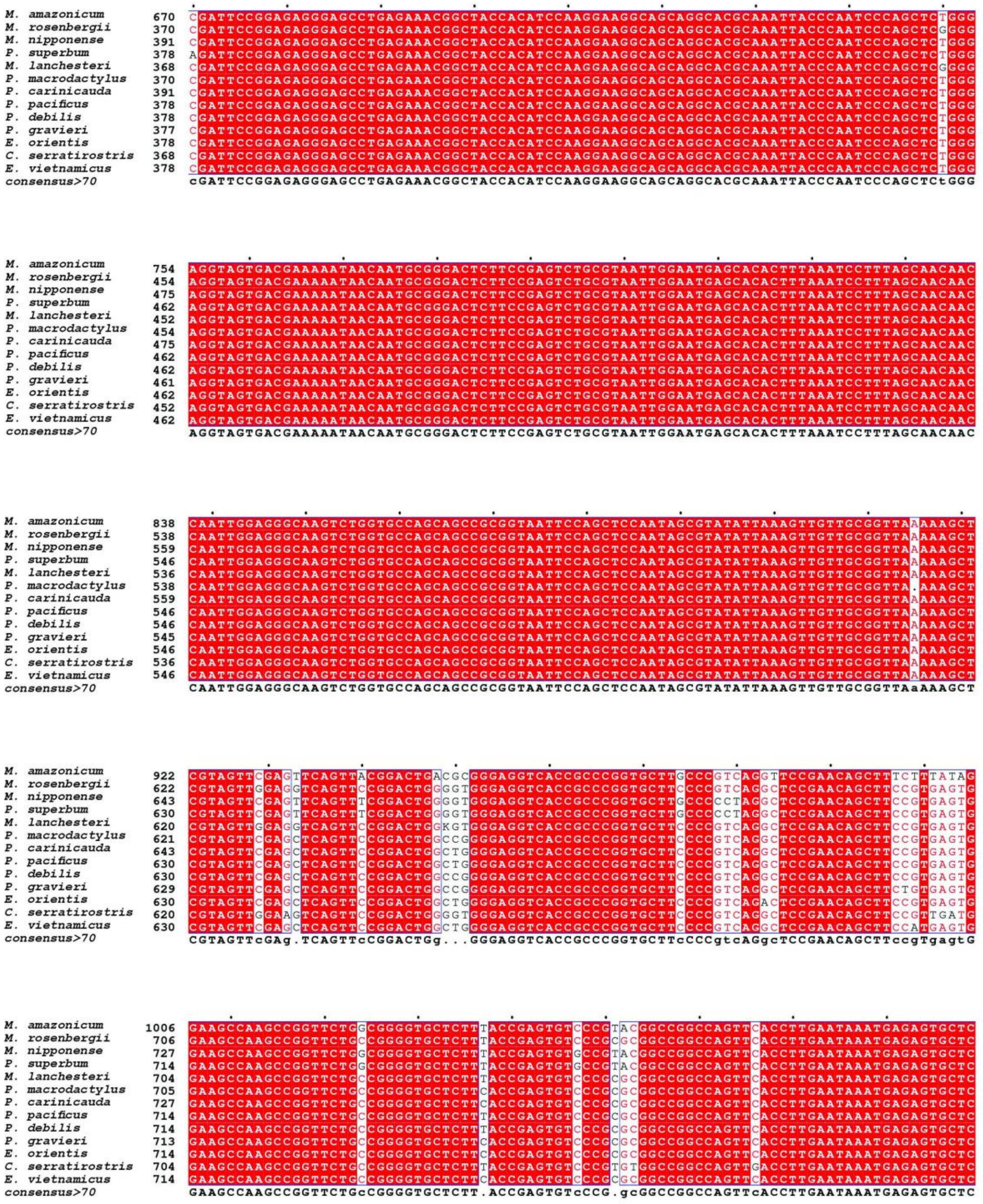

Figure S1B. 18S gene continued.

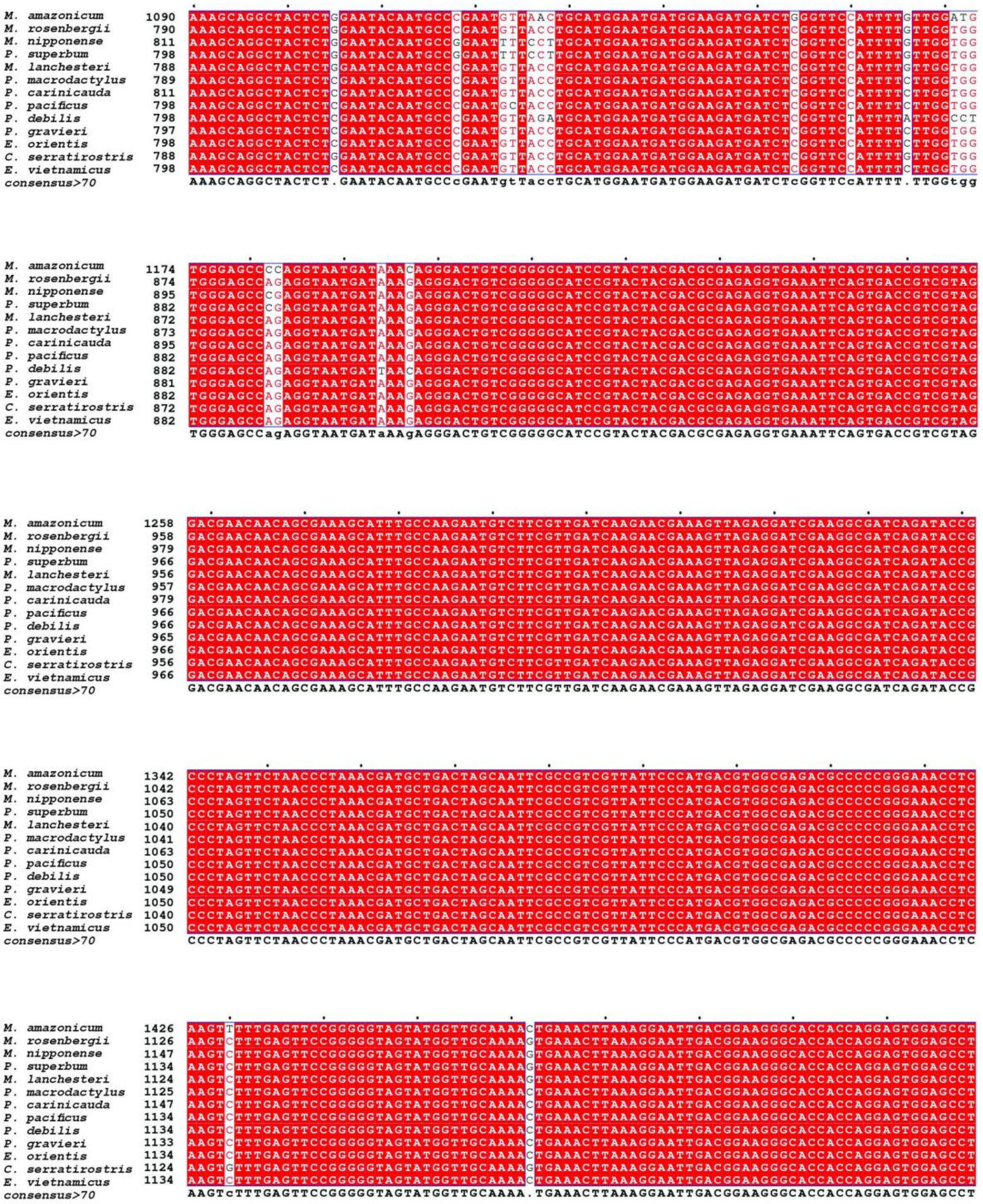

Figure S1B. 18S gene continued.

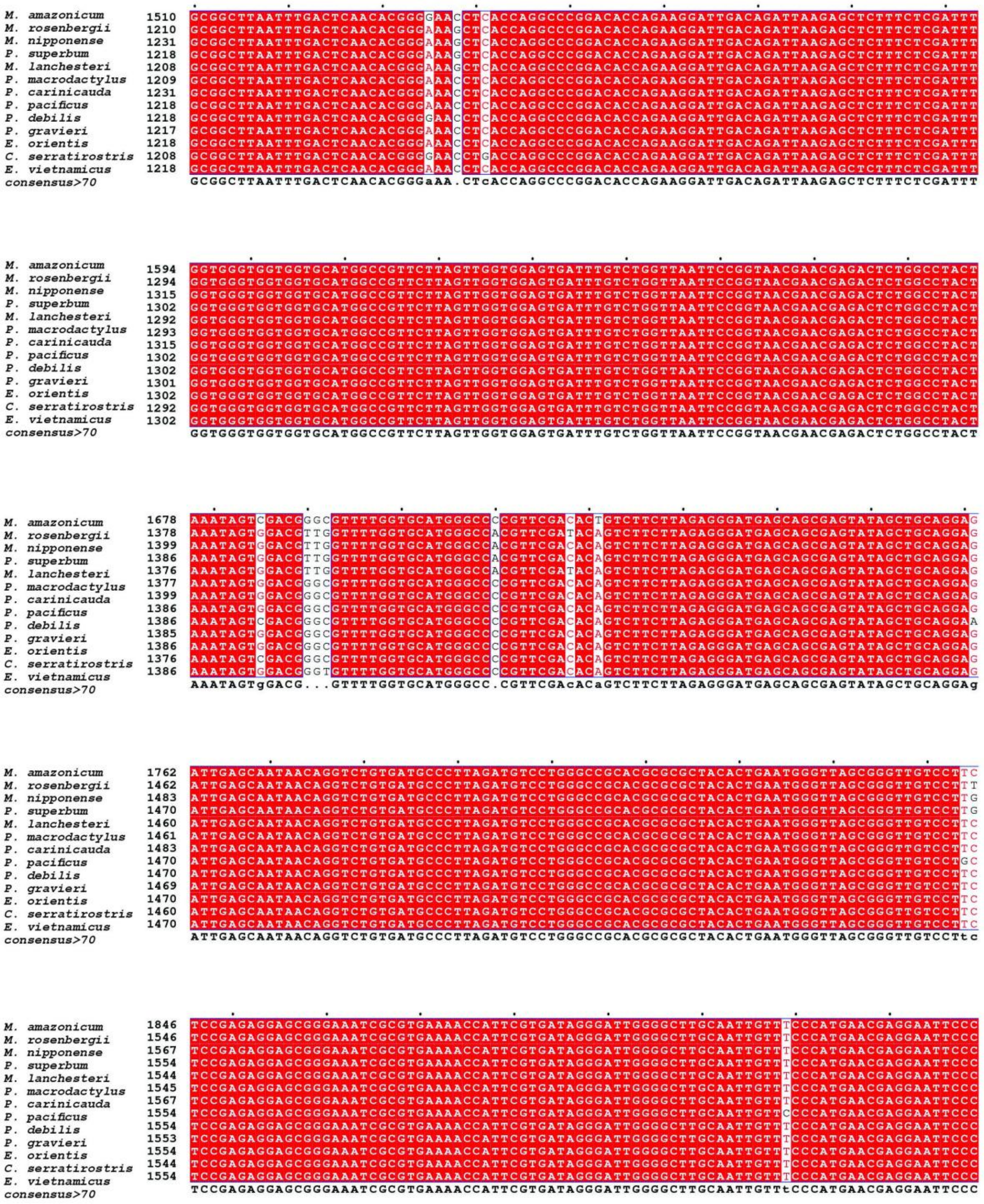

Figure S1B. 18S gene continued.

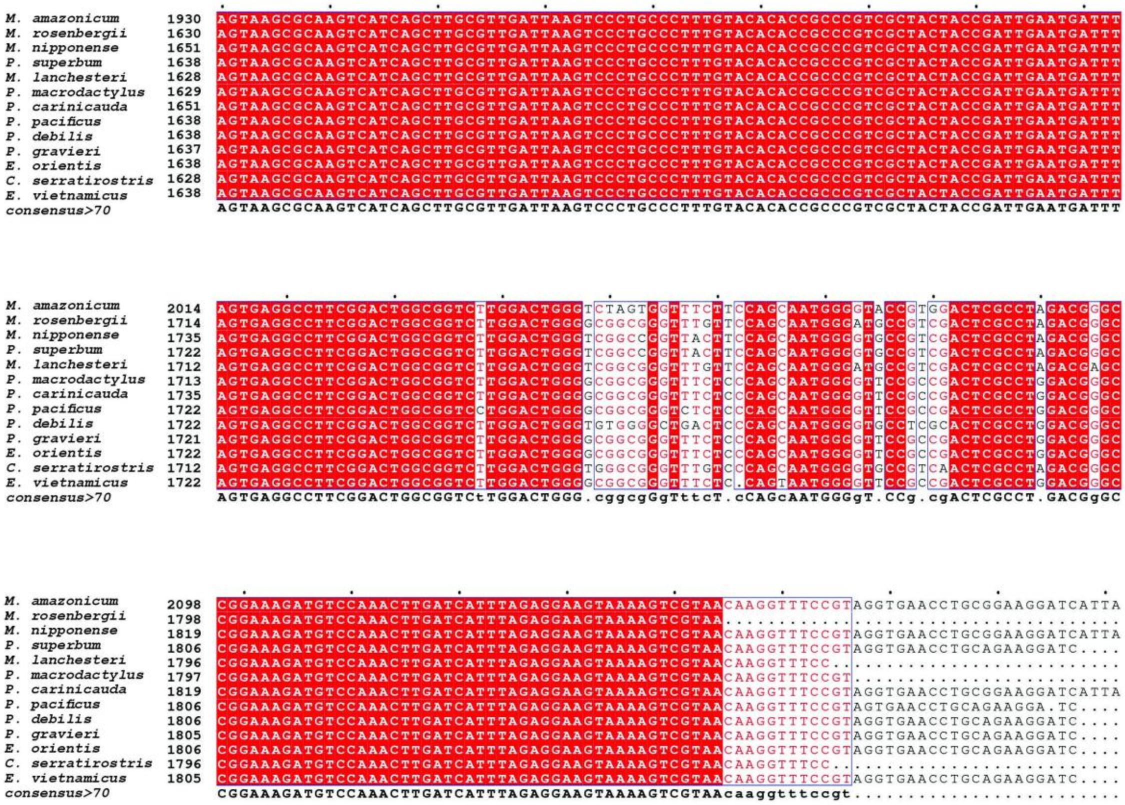

Figure S1C. RPL18 gene.

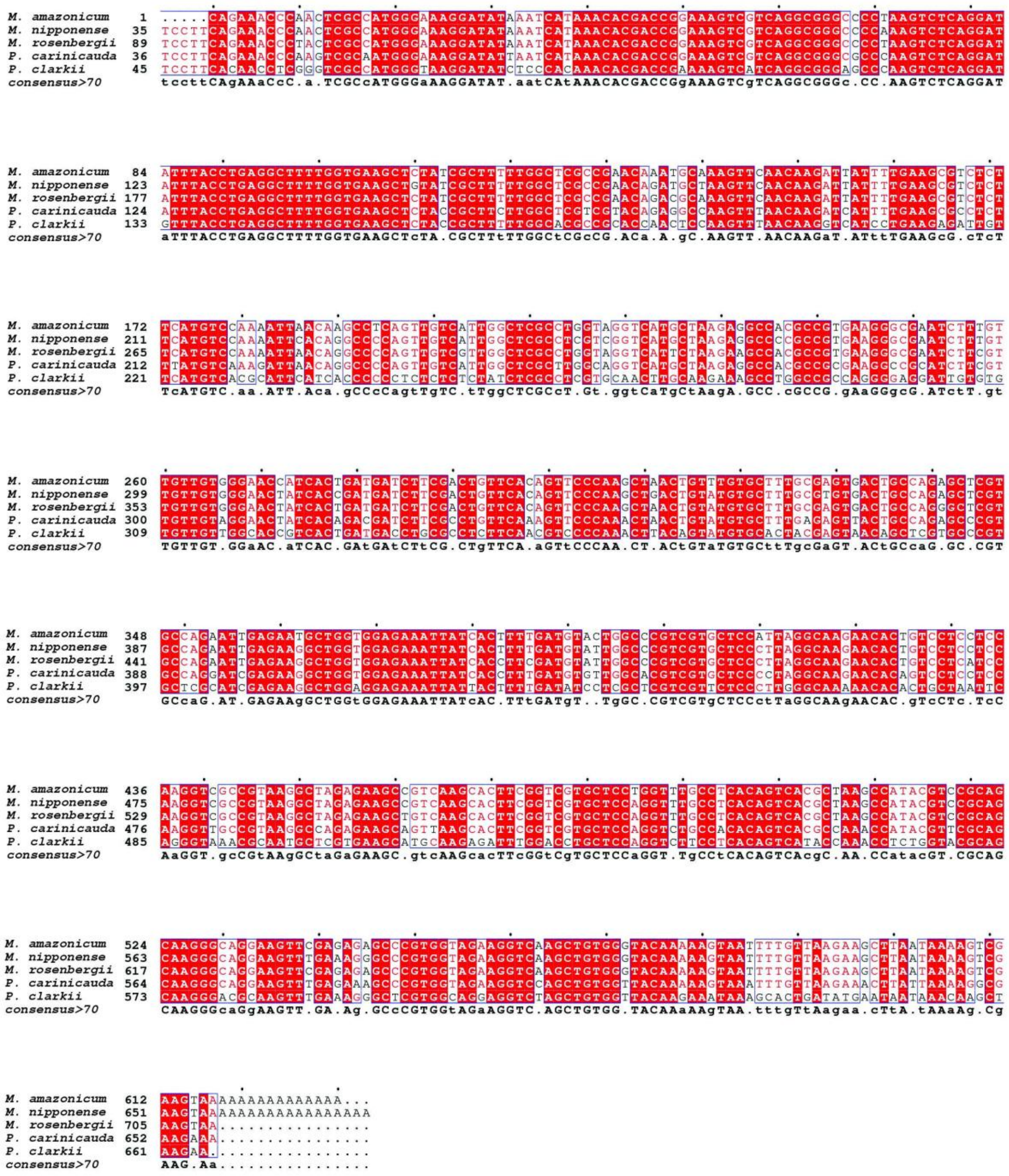

Figure S1D. β-actin gene.

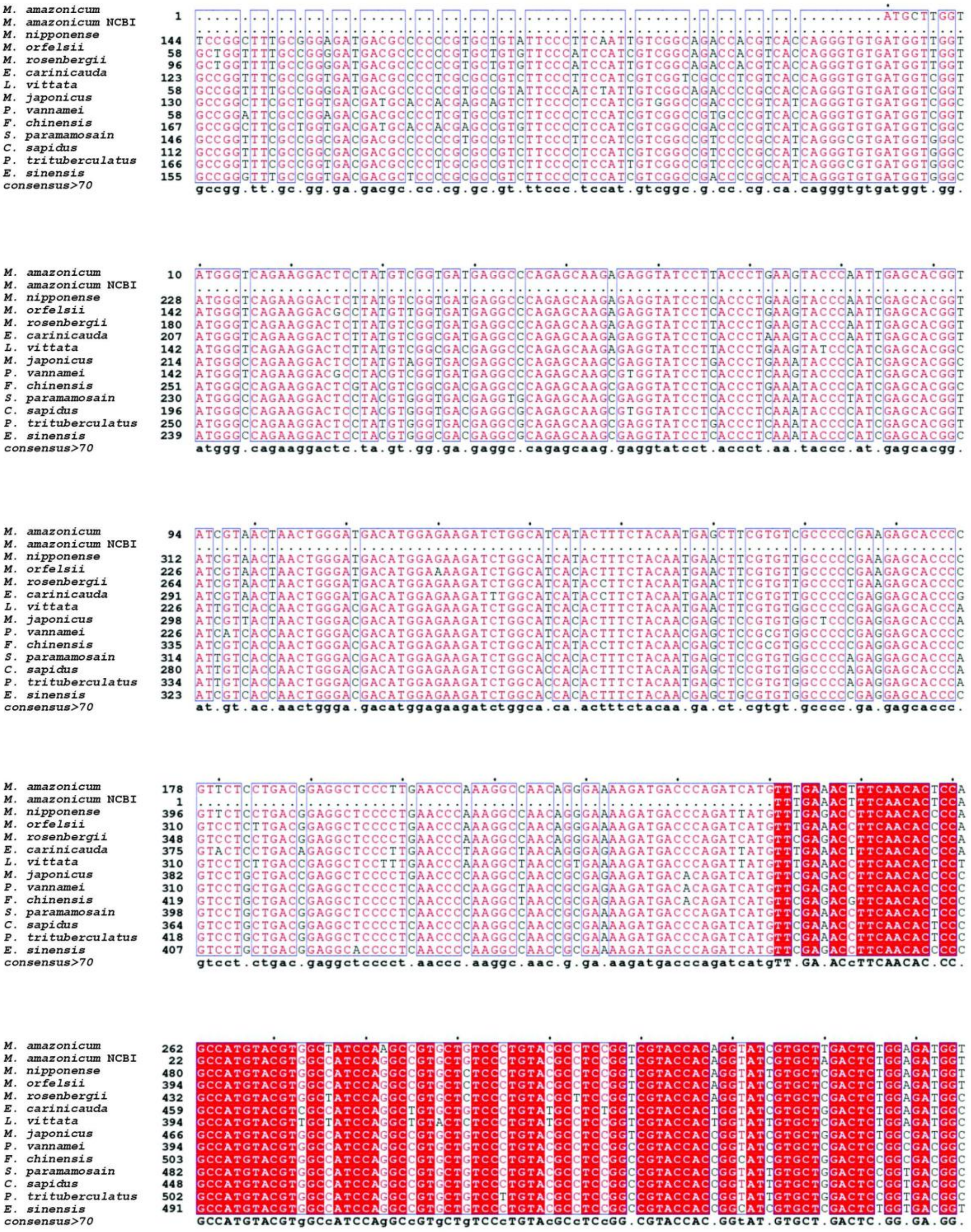

Figure S1D. β-actin gene continued.

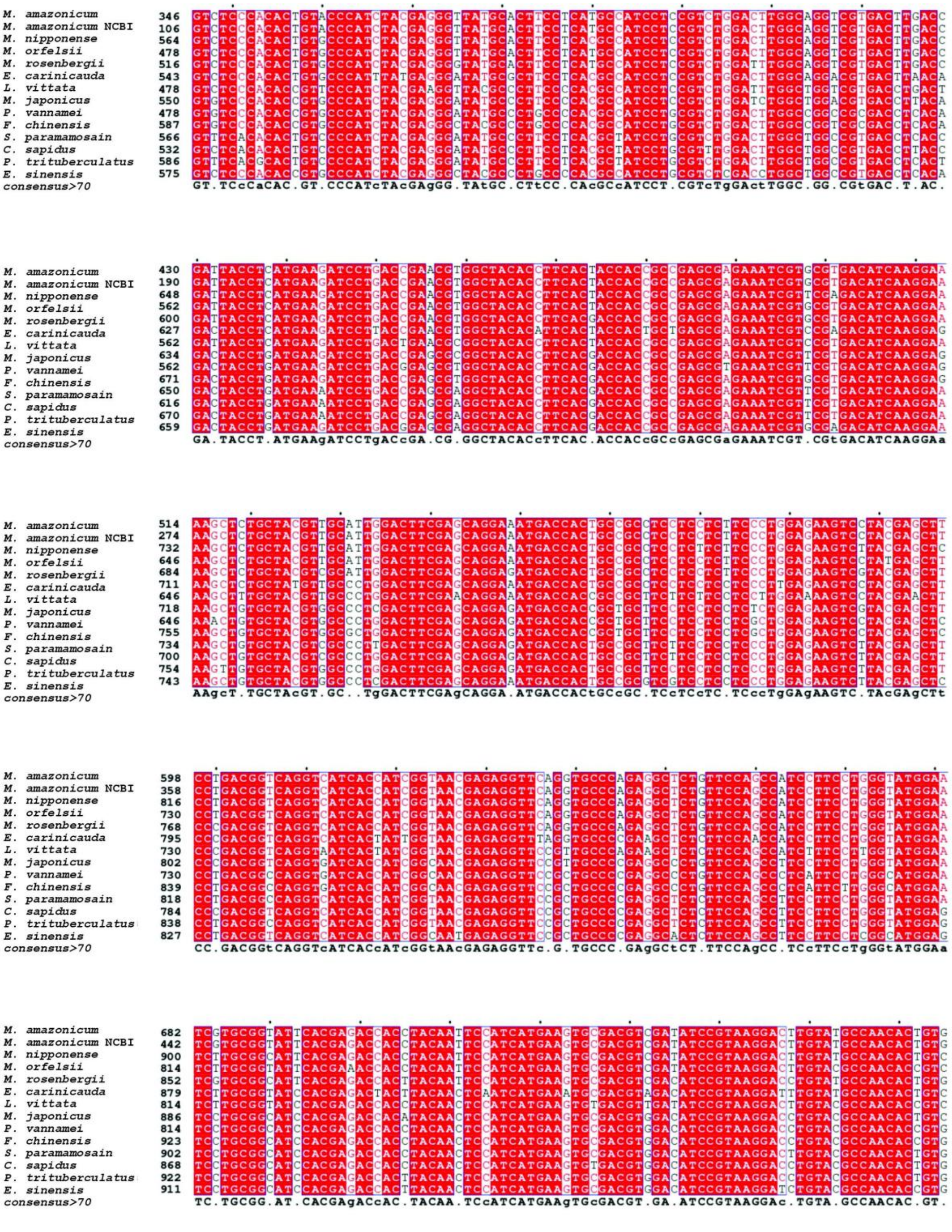

Figure S1D. β-actin gene continued.

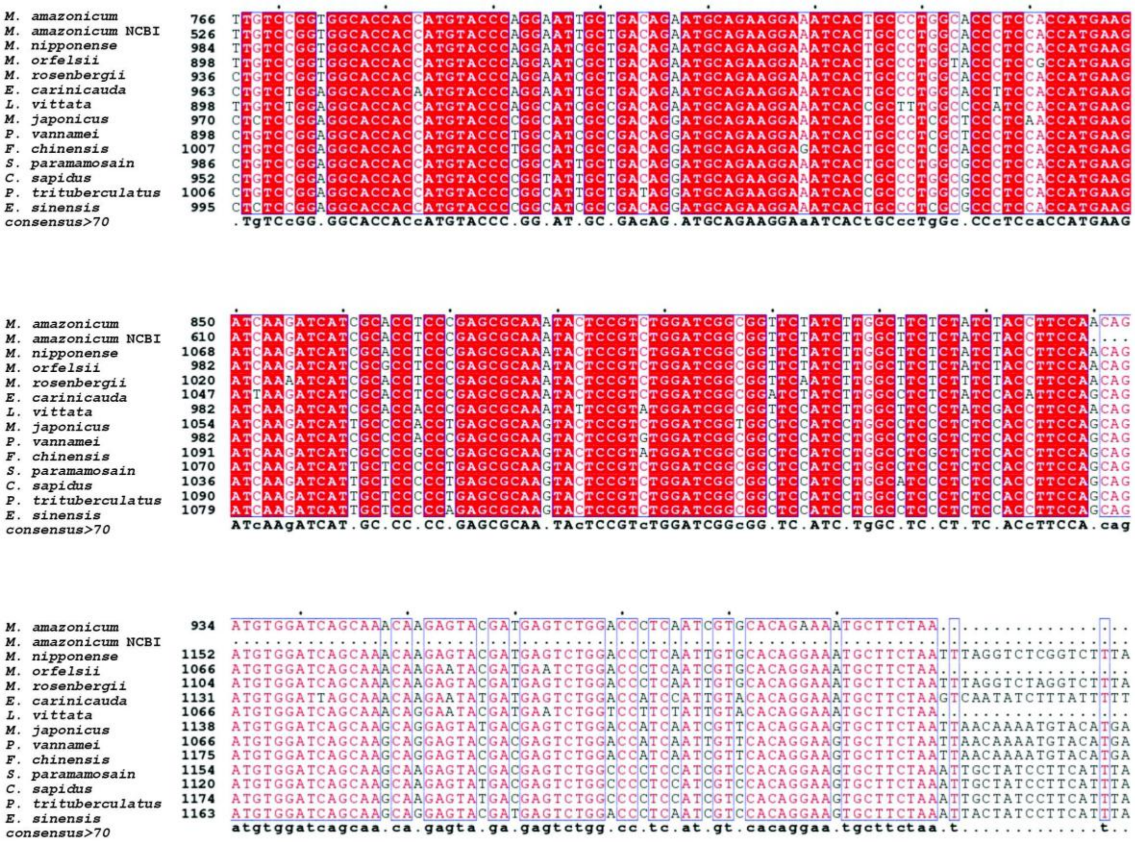

Figure S1E. α-tub gene.

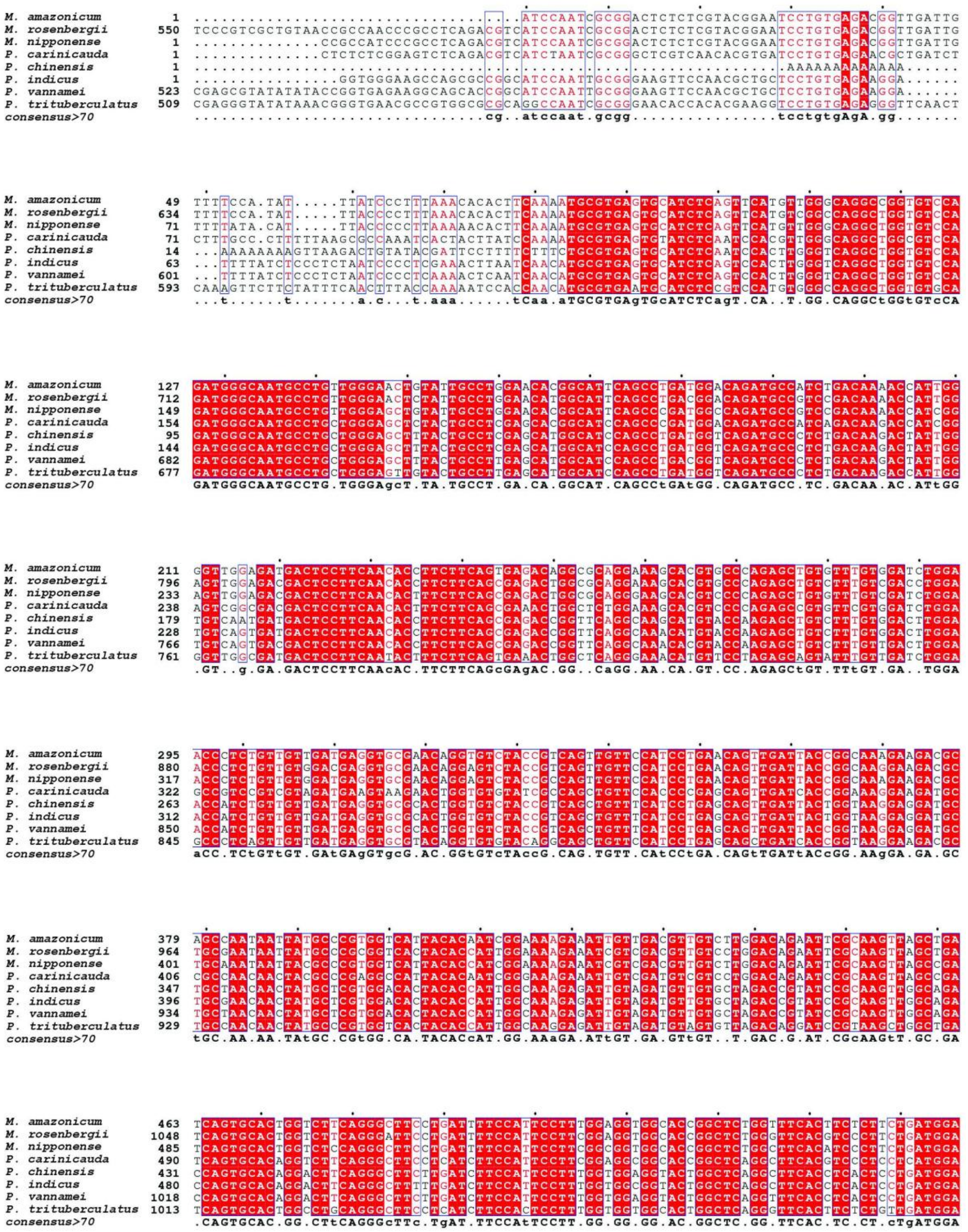

Figure S1E. α-tub gene continued.

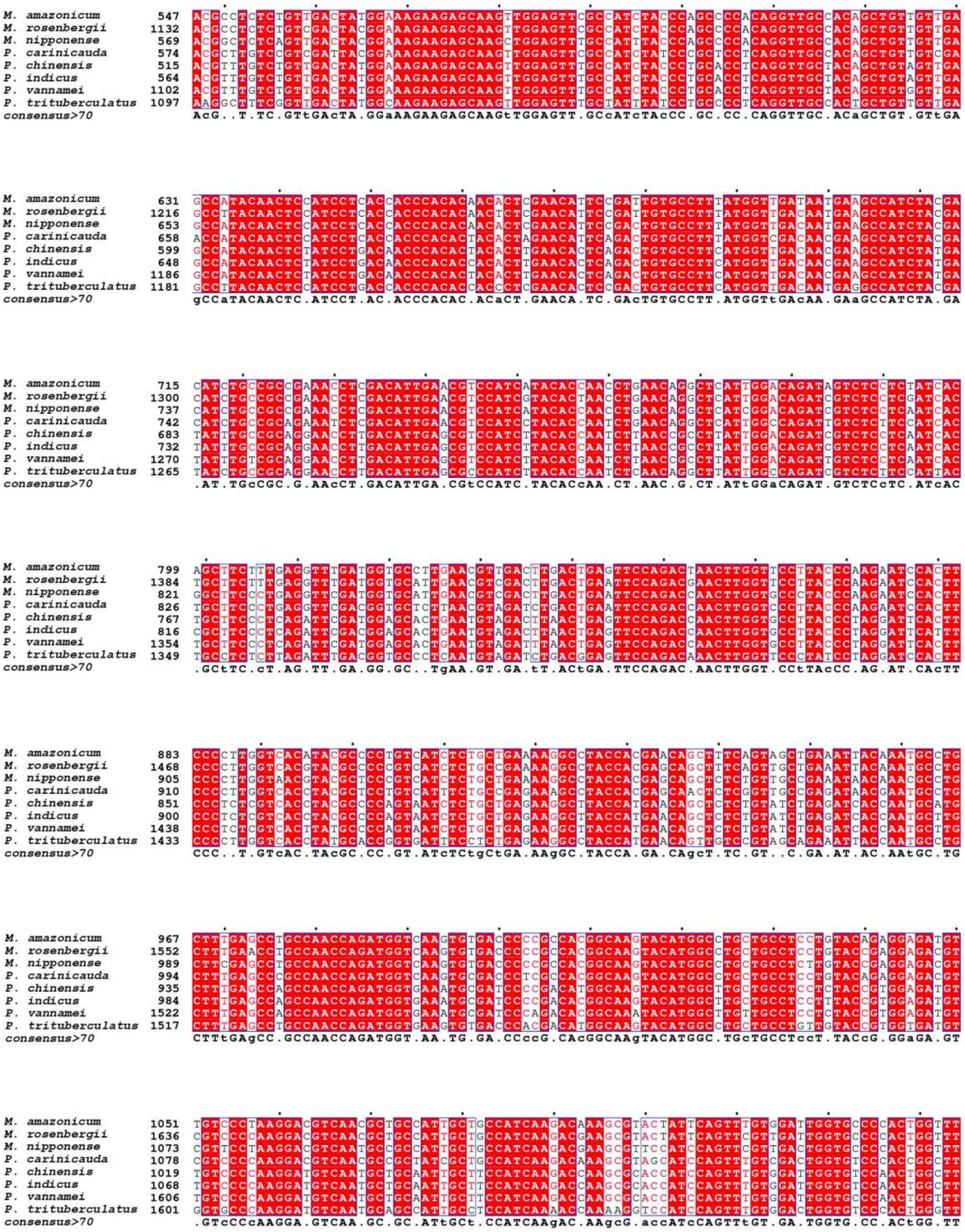

Figure S1E. α-tub gene continued.

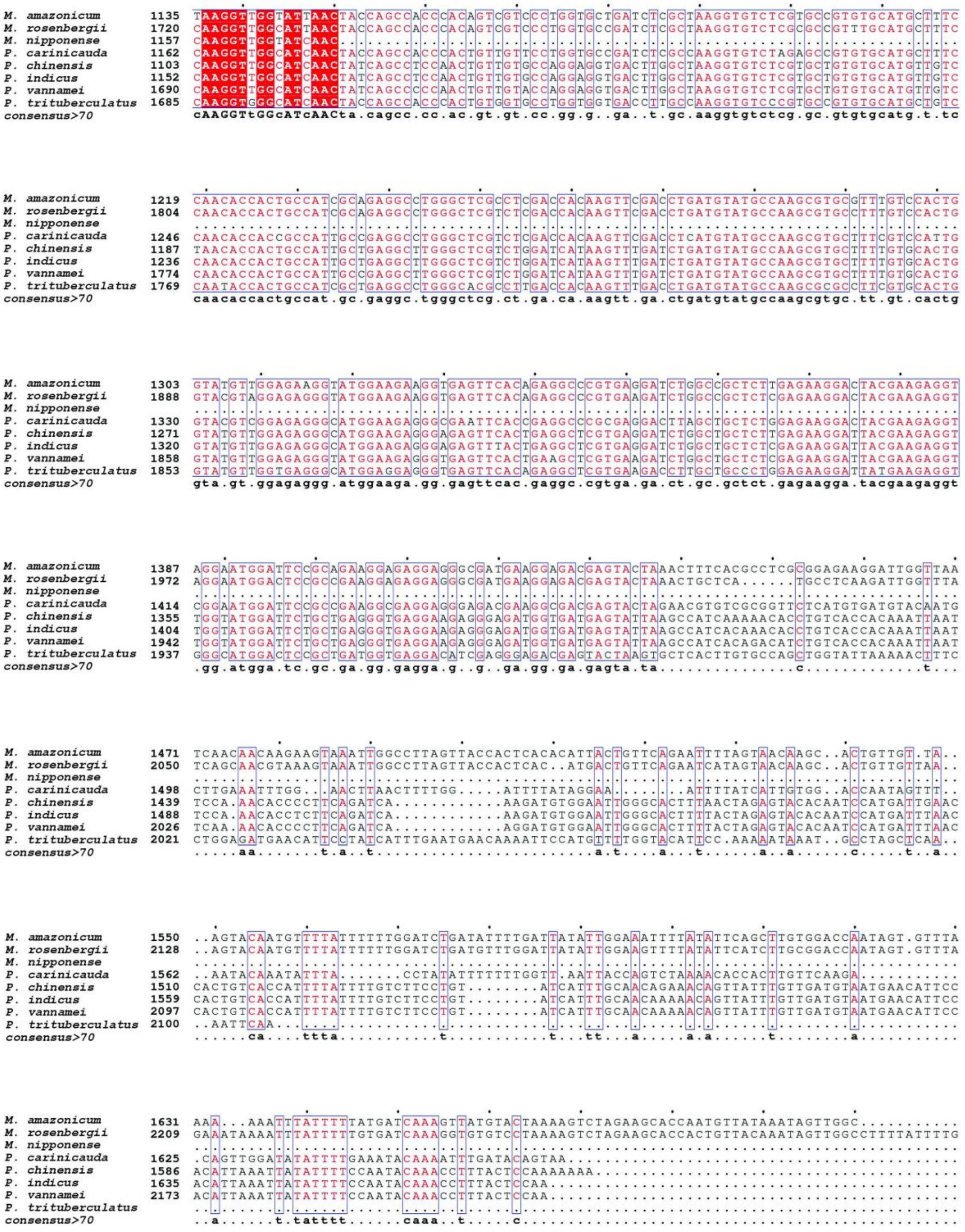

Figure S1F. EF1-α gene.

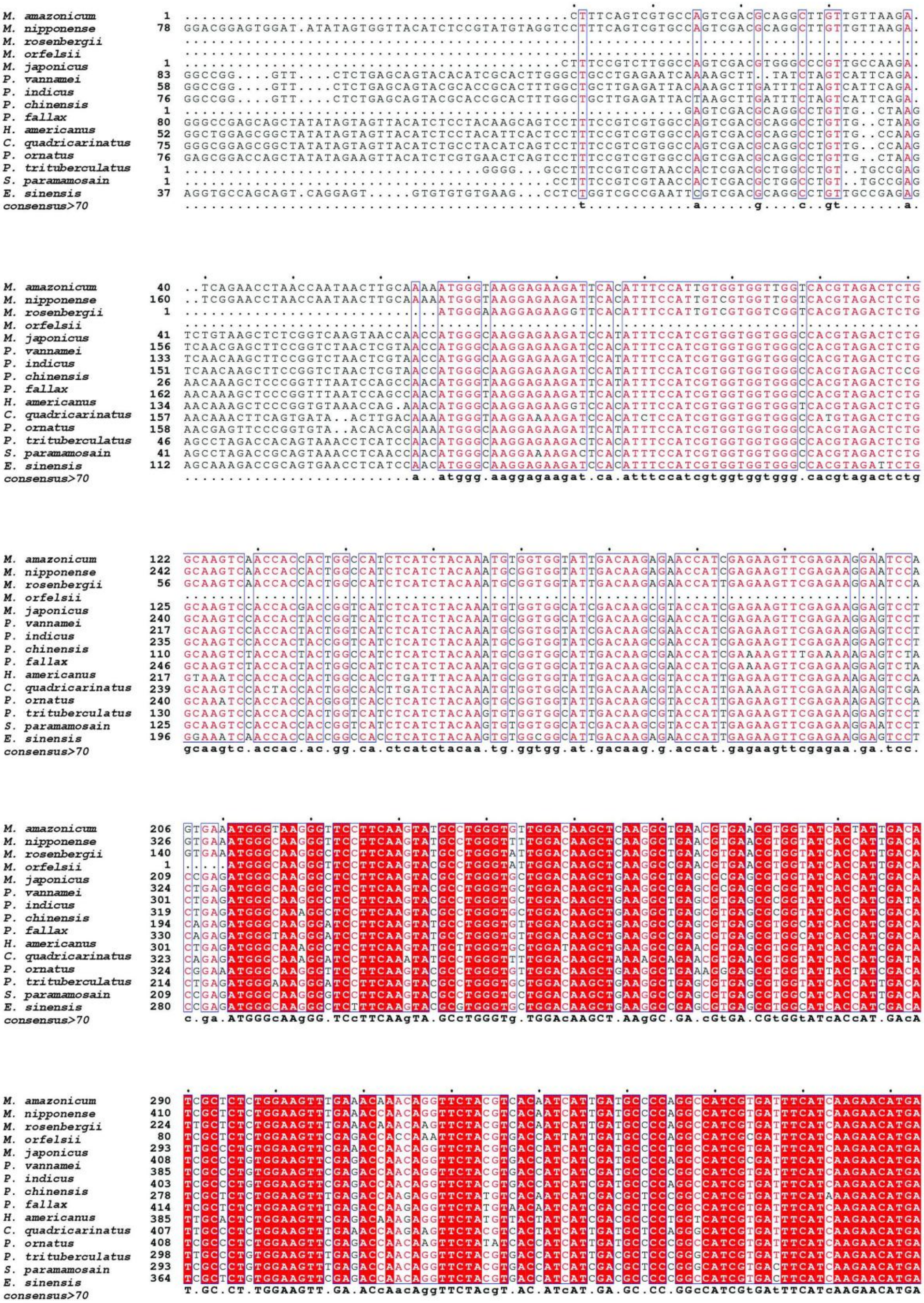

Figure S1F. EF1-α gene continued.

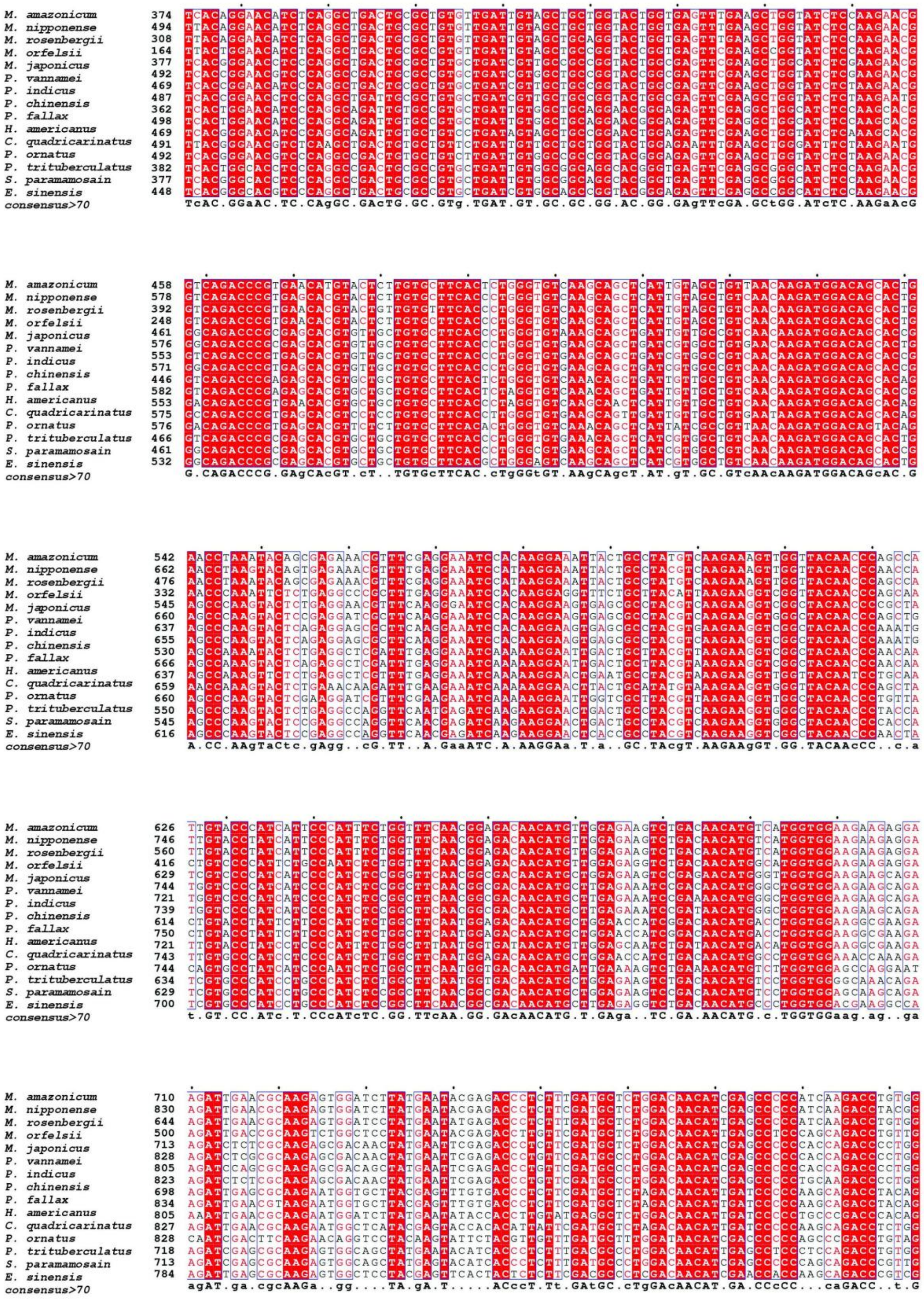

Figure S1F. EF1-α gene continued.

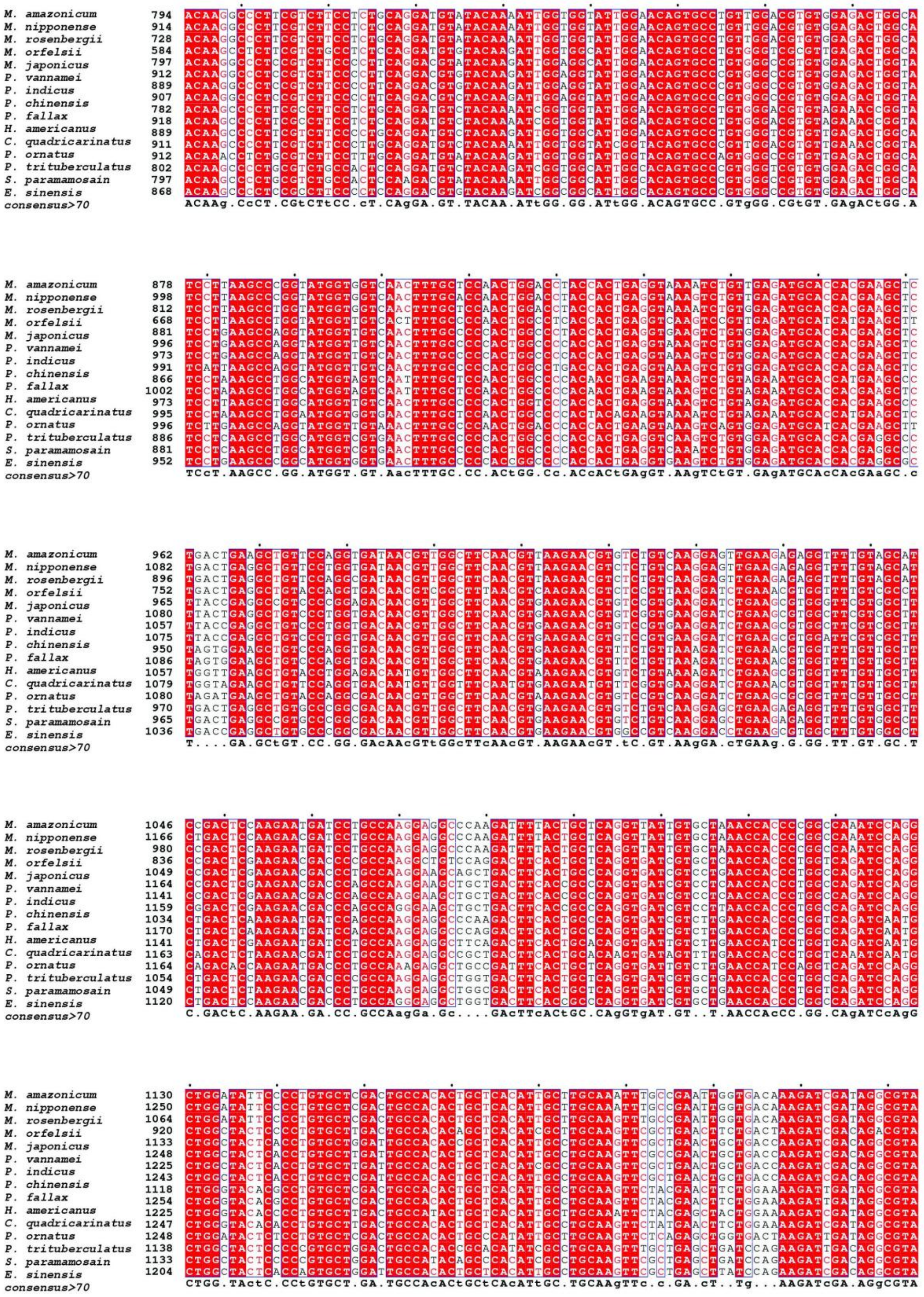

Figure S1F. EF1-α gene continued.

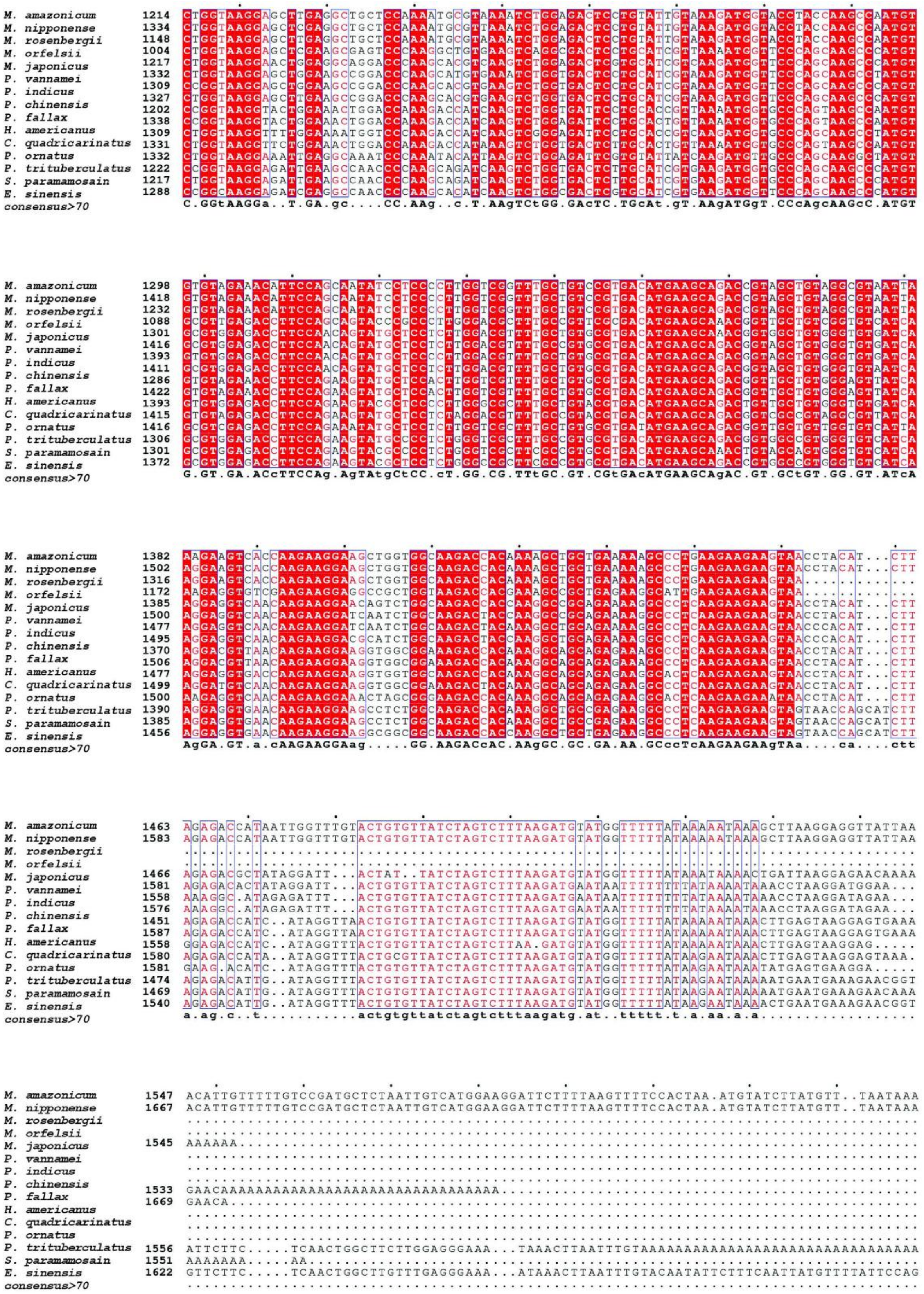

Figure S1G. GAPDH gene.

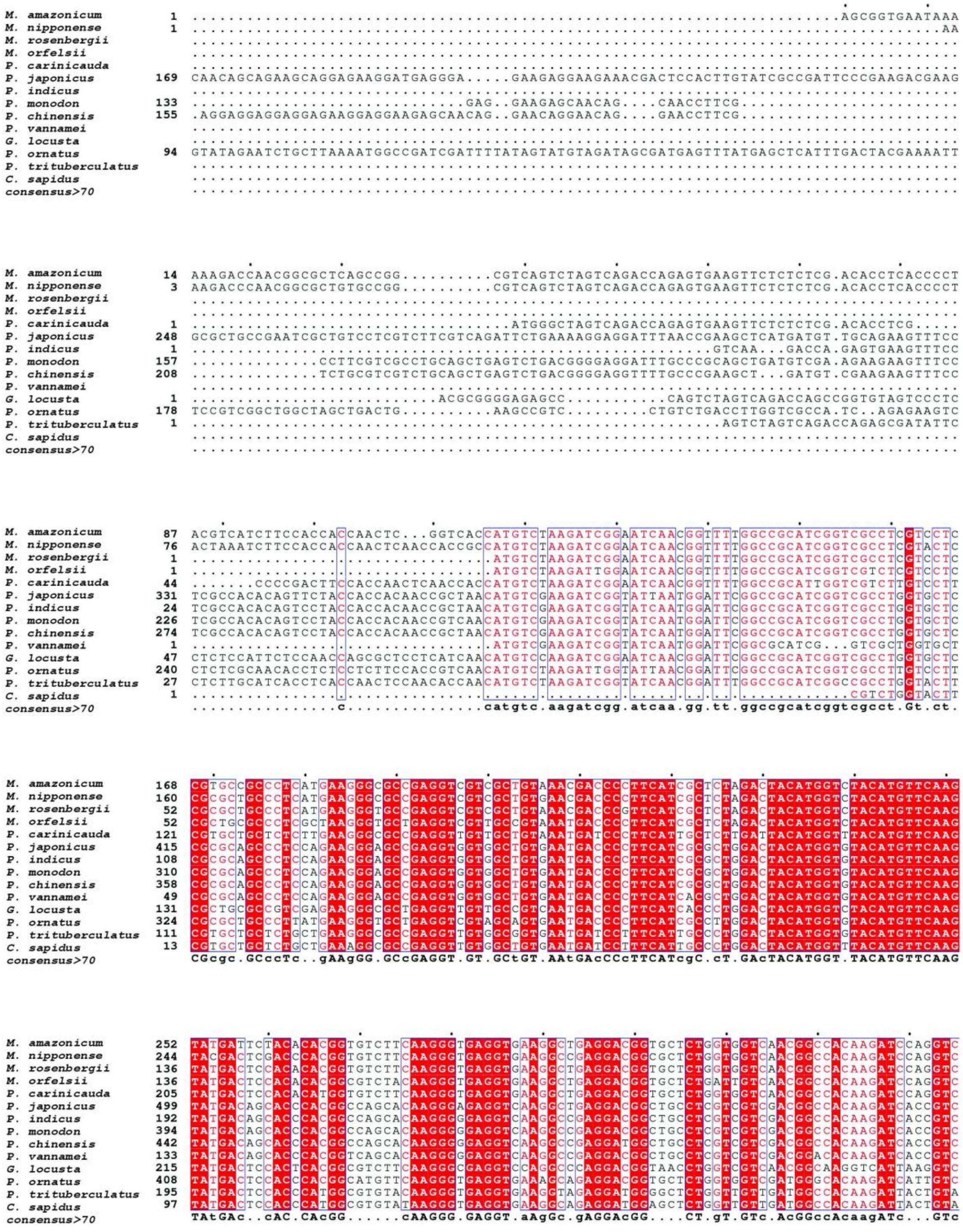

Figure S1G. GAPDH gene continued.

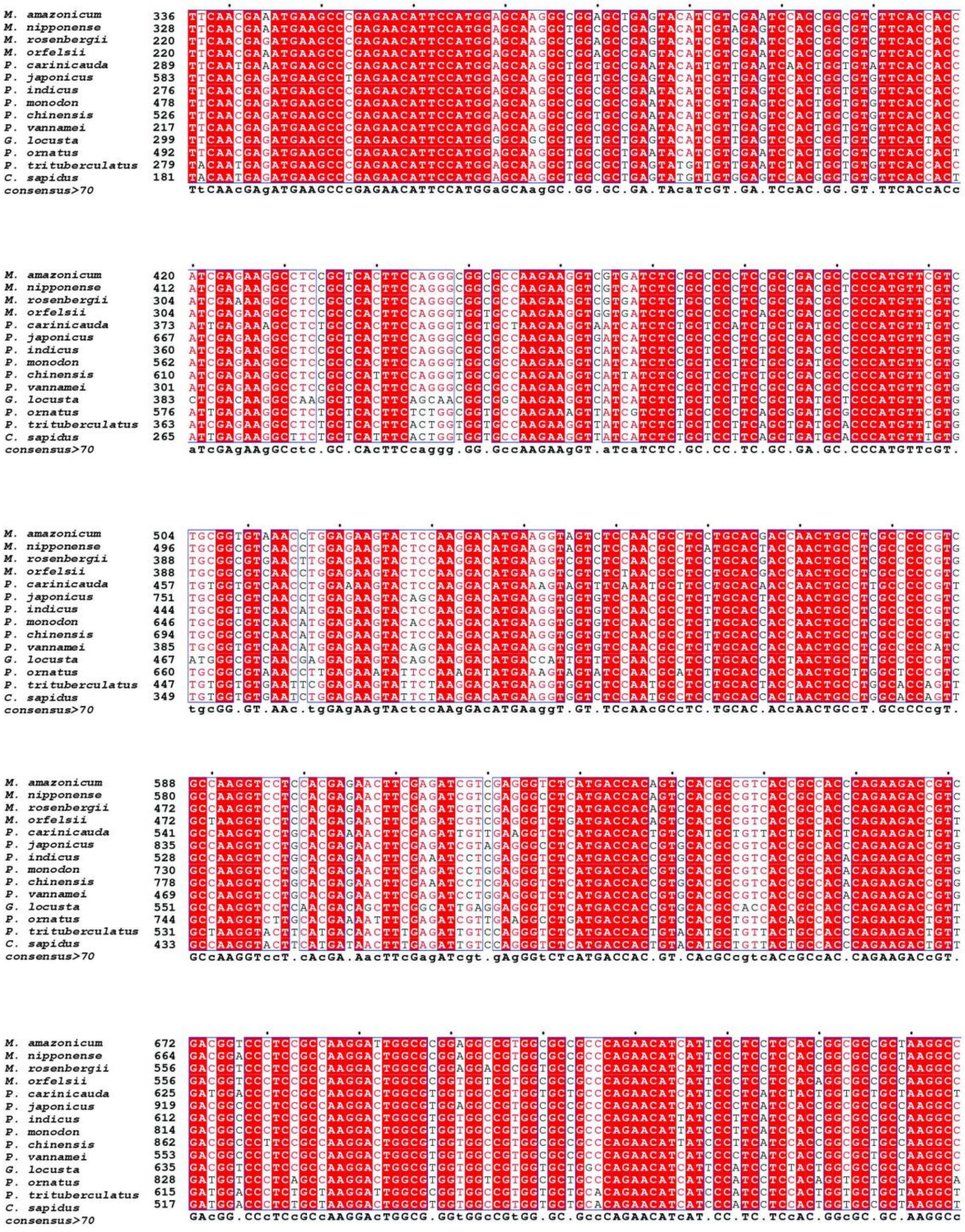

Figure S1G. GAPDH gene continued.

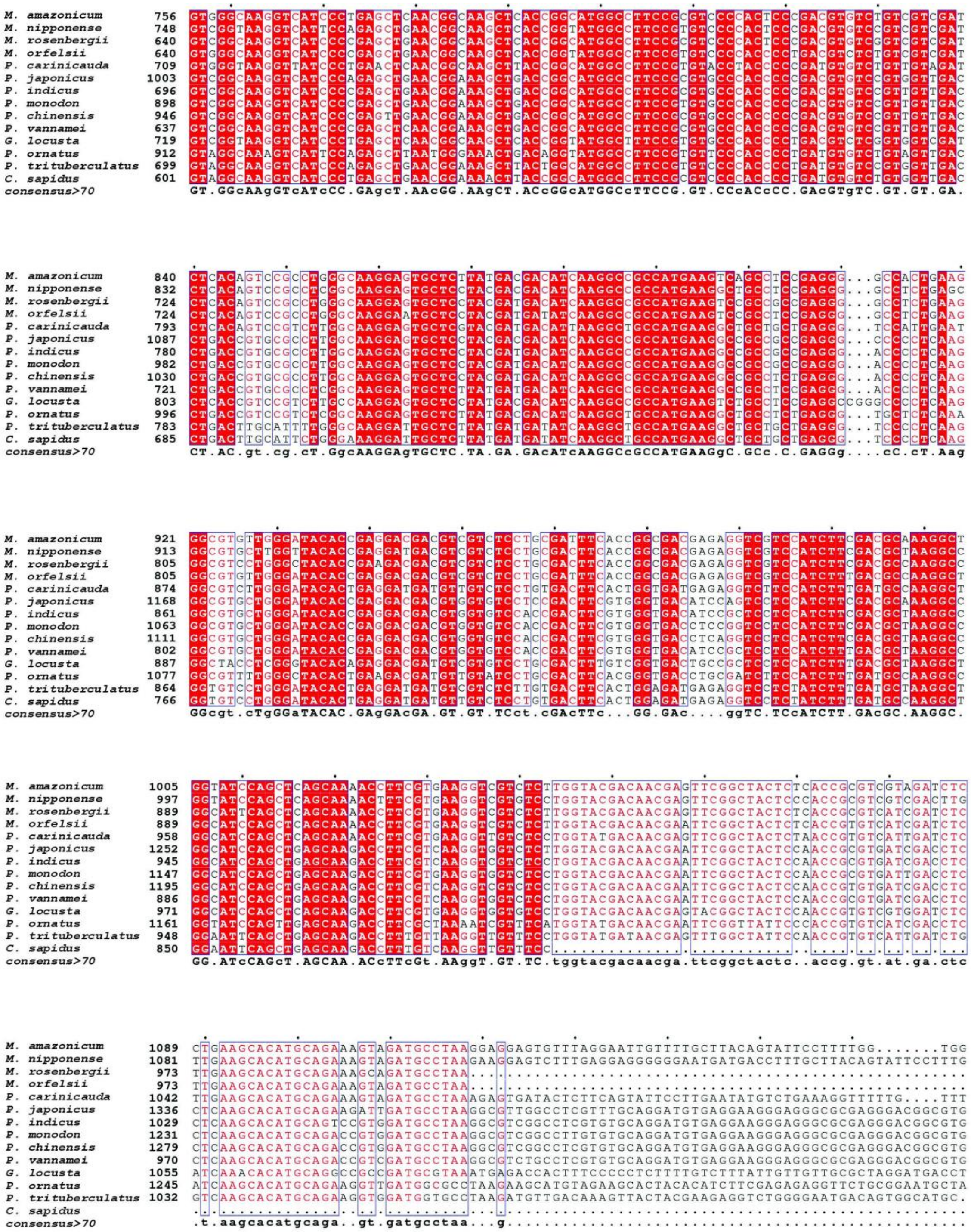

Figure S1G. GAPDH gene continued.

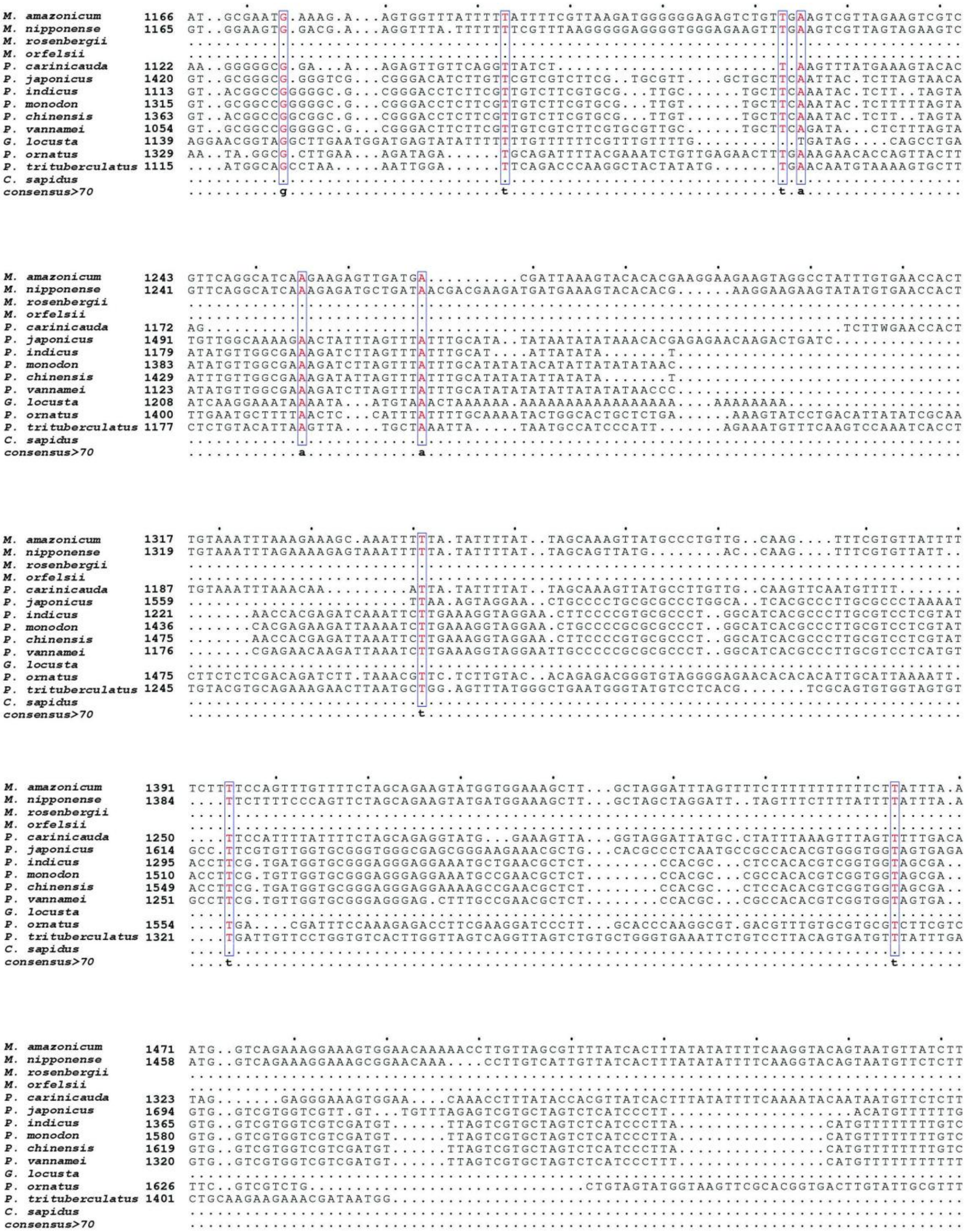

Figure S1G. GAPDH gene continued.

```
M. amazonicum        1553 TGTGCTAGTCTTACAAACCCCGTTGTCTTTGTTGTCTTAATAAGAAG....TAATGTAAGTTGACCGACTGGATGGCCAATAAA
M. nipponense        1536 TGTGCTAGTCCTACAAACCCTATTGTCTTTGTTGTCTTTAAGAAGTATGTAATGTATAAGTTGACCGACTGGATGGCCAATAAA
M. rosenbergii            ....................................................................................
M. orfelsii               ....................................................................................
P. carinicauda       1396 TGTGCTAGTCTACAA..CCCCAATGTCTTTGTTGTCTTTAAG......AAGTAATGTAAGTTGACCGACTGGATGGCCAATAAA
P. japonicus         1752 TCTGATAAACACTC......................TGGATCAATGATGTATGCTATTTGACCAACTGGATATTGGCCAATAAA
P. indicus           1425 TGAAACACTCTGGATCAAT...........GATG..............TATGCTACTTTGACCAACTGGATATTGGCCAATAAA
P. monodon           1640 TGAAACACTCTGGATCAAT...........GATG..............TATGCTACTTTGACCAACTGGATATTGGCCAATAAA
P. chinensis         1679 TGAAACACTCTGGATCAATGATGGATCAATGATG..............TATGCTACTTTGACCAACTGGATATTGGCCAATAAA
P. vannamei          1380 TTTTGTC....TGAAACACTCTGGATCAATGATG..............TATGCTACTTTGACCAACTGGATATTGGCCAATAAA
G. locusta                ....................................................................................
P. ornatus           1678 CGTGCAAGTGGGTATGACTCTTGTATCAGT..................GTGTTGGTGAAGACCAACTGGATATAGGCCAATAAA
P. trituberculatus   1423 ............................................................................CCAATAAA
C. sapidus                ....................................................................................
consensus>70              ............................................................................ccaataaa
```

```
M. amazonicum        1633 AAGATTTATTTCAAAAAAAA...........................
M. nipponense        1620 AAGATTTATTTCAAAAAAAAAAAAAAAAAAAA...............
M. rosenbergii            ...............................................
M. orfelsii               ...............................................
P. carinicauda       1472 AAGATTTATTTCAAAAAAAAAAAAAAAAAAAAAAAAAAAAAAA....
P. japonicus         1814 GAACTTAATTTCA..................................
P. indicus           1484 GAACTTAATTTCACACAA.............................
P. monodon           1699 GAACTTAATTTCA..................................
P. chinensis         1749 GAACTTAATTTCA..................................
P. vannamei          1446 GAACTTAATTACGGAAAAAAAAAAAAAAAAAAAAAAAAAAAAAAAAA
G. locusta                ...............................................
P. ornatus           1744 AGACTAAAAATTCC..................AA.............
P. trituberculatus   1431 AGAATCTGTTACAGAAAAAAAAAAGAA....................
C. sapidus                ...............................................
consensus>70              ....t..........................................
```
