# Supplementary material for: Identification and Validation of Tissue-Specific Housekeeping Markers for the Amazon River Prawn Macrobrachium amazonicum (Heller, 1862)
Source: Genes (Basel). 2025 Dec 28;17(1):26. doi: 10.3390/genes17010026 (PMC12840830; doi:10.3390/genes17010026)
Supplement: Supplementary file 1 [file genes-17-00026-s001.zip › Table S1.pdf]

Table S1. Homologous crystal structure models employed for the tree-dimensional modeling of *Macrobrachium amazonicum* housekeeping gene (HKG) candidates. The file reports the structural similarity (%) of the templates, the corresponding species, accession numbers, and the respective references, when available. * Evaluation of amino acid (aa) similarity. ** Specific taxon of the species not defined.

**Gene**	**Structural similarity ***	**Template species**	**Accession number**	**Reference**
EIF	90.4	*Portunus trituberculatus*	A0A5B7E1N1.1	Unpublished
RPL18	68.6	*Mammalian* **	7zjw.18	[88]
β-actin	98.8	*Drosophila* sp.	8oh4.1	[89]
α-tub	96.6	*Homarus americanus*	Q25008.1	[90]
EF1-α	79.5	*Oryctolagus cuniculus*	6ra9.1	[91]
GAPDH	94.2	*Palinurus versicolor*	1crw.1	[92]
