# Supplementary material for: Identification and Validation of Tissue-Specific Housekeeping Markers for the Amazon River Prawn Macrobrachium amazonicum (Heller, 1862)
Source: Genes (Basel). 2025 Dec 28;17(1):26. doi: 10.3390/genes17010026 (PMC12840830; doi:10.3390/genes17010026)
Supplement: Supplementary file 1 [file genes-17-00026-s001.zip › Table S2.pdf]

Table S2. Summary of the stability tests for the HKG candidates in the different tissues of *Macrobrachium amazonicum*.

Tissue	Method	Genes Rank (Better - Good - Average)						
		1°	2°	3°	4°	5°	6°	7°
All tissues	Comparative ΔCt	RPL18	β-actin	18S	EIF	α-tub	EF1-α	GAPDH
	BestKeeper	18S	RPL18	EIF	β-actin	EF1-α	α-tub	GAPDH
	NormFinder	RPL18	18S	EIF	β-actin	α-tub	EF1-α	GAPDH
	GeNorm	18S ǀ RPL18		EIF	β-actin	α-tub	EF1-α	GAPDH
	R. comprehensive ranking	RPL18	18S	EIF	β-actin	α-tub	EF1-α	GAPDH
Muscle	Comparative ΔCt	EF1-α	RPL18	β-actin	18S	α-tub	EIF	GAPDH
	BestKeeper	EIF	α-tub	β-actin	EF1-α	RPL18	18S	GAPDH
	NormFinder	RPL18	EF1-α	β-actin	18S	α-tub	EIF	GAPDH
	GeNorm	β-actin ǀ α-tub		EF1-α	EIF	RPL18	18S	GAPDH
	R. comprehensive ranking	EF1-α	β-actin	RPL18	α-tub	EIF	18S	GAPDH
Hepatopancreas	Comparative ΔCt	α-tub	EIF	RPL18	18S	EF1-α	β-actin	GAPDH
	BestKeeper	18S	EIF	RPL18	β-actin	α-tub	EF1-α	GAPDH
	NormFinder	α-tub	EIF	RPL18	18S	EF1-α	β-actin	GAPDH
	GeNorm	EIF ǀ 18S		RPL18	α-tub	EF1-α	β-actin	GAPDH
	R. comprehensive ranking	EIF	18S	α-tub	RPL18	EF1-α	β-actin	GAPDH
Gills	Comparative ΔCt	RPL18	EIF	β-actin	EF1-α	18S	α-tub	GAPDH
	BestKeeper	18S	RPL18	EF1-α	EIF	α-tub	β-actin	GAPDH
	NormFinder	RPL18	EIF	β-actin	EF1-α	18S	α-tub	GAPDH
	GeNorm	EIF ǀ β-actin		RPL18	EF1-α	18S	α-tub	GAPDH
	R. comprehensive ranking	RPL18	EIF	β-actin	18S	EF1-α	α-tub	GAPDH
Testis	Comparative ΔCt	α-tub	EIF	18S	RPL18	GAPDH	β-actin	EF1-α
	BestKeeper	RPL18	18S	α-tub	EIF	EF1-α	GAPDH	β-actin
	NormFinder	EIF	α-tub	18S	RPL18	GAPDH	β-actin	EF1-α
	GeNorm	EIF ǀ α-tub		18S	RPL18	GAPDH	β-actin	EF1-α
	R. comprehensive ranking	α-tub	EIF	18S	RPL18	GAPDH	β-actin	EF1-α
Androgenic gland	Comparative ΔCt	18S	EIF	β-actin	α-tub	EF1-α	RPL18	GAPDH
	BestKeeper	18S	RPL18	GAPDH	α-tub	EIF	β-actin	EF1-α

	NormFinder	18S	EIF	α-tub	β-actin	EF1-α	RPL18	GAPDH
	GeNorm	EIF ∣ β-actin		EF1-α	α-tub	18S	RPL18	GAPDH
	R. comprehensive ranking	18S	EIF	β-actin	α-tub	RPL18	EF1-α	GAPDH
	Comparative ΔCt	α-tub	β-actin	EIF	GAPDH	RPL18	EF1-α	18S
	BestKeeper	18S	RPL18	β-actin	α-tub	EIF	GAPDH	EF1-α
Ovary	NormFinder	β-actin	α-tub	EIF	GAPDH	RPL18	EF1-α	18S
	GeNorm	EIF ∣ GAPDH		EF1-α	α-tub	β-actin	RPL18	18S
	R. comprehensive ranking	β-actin	α-tub	EIF	GAPDH	RPL18	18S	EF1-α
